# Anion–π interactions influence p*K*_a_ values

**DOI:** 10.3762/bjoc.7.42

**Published:** 2011-03-17

**Authors:** Christopher J Cadman, Anna K Croft

**Affiliations:** 1 School of Chemistry, University of Wales Bangor, Bangor, Gwynedd, LL57 2UW, United Kingdom. Fax: +44 1248 370 528. Tel: +44 1248 382 375

**Keywords:** anion–π, DFT, intramolecular interaction, LFER, p*K*_a_

## Abstract

Five 8-(4-R-phenyl)-1-naphthol derivatives were prepared by PdCl_2_-catalysed electrophilic aromatic substitution. The p*K*_a_' values for these 1,8-disubstituted arene naphthols have been measured in acetonitrile/water (R = NO_2_, 8.42; R = Cl, 8.52; R = H, 8.56; R = Me 8.68; and R = OMe, 8.71) and indicate a correlation with the electronic nature of the arene substituent, as determined through LFER analysis. Contributions to the relative p*K*_a_' values have been interpreted, using M06-2X DFT calculations, as consisting of two components: A small contribution from initial OH–π bonding in the starting materials and a larger contribution from anion–π interactions in the products. Such effects have implications for a range of other systems.

## Introduction

There are numerous examples in nature of interactions involving aromatic systems and these interactions underpin many modern supramolecular binding agents, with clear applications in biological, medical and environmental chemistry [1]. Cation–π and π–π interactions are perhaps the best known of these non-covalent forces and are driven by attractions between the quadrupole moments of the aromatic species in question, with either a cation or other aromatic, respectively. In a similar fashion, CH–π interactions and anion–π interactions have been identified as influencing binding in a number of systems. Since key computational investigations have indicated that anion–π interactions might be very important [2–4], which is also supported by strong circumstantial evidence from crystal-structure mining [5–7], there has been a resurgence of work in this area.

One prime focus has been on anion–π interactions as a means to design selective supramolecular anion receptors and template-directed synthesis of macrocyclic complexes has also been achieved [8–17]. The magnitude of the anion–π interaction varies with the size of the aromatic quadrupole and the polarisability of the system. Recent quantitative measurements of chloride binding to calixarenes in solution estimate these interactions to be as much as 4.6 kJ·mol^−1^ [18]. Moreover, computational models suggest that these interactions can be further enhanced through co-operative interactions [19–20].

In addition to guiding binding interactions, aromatic groups are also able to direct reaction outcomes. This has been well established in the field of cation–π interactions, where early experiments by Cram indicated that aromatics in close proximity to an incipient carbocation could accelerate tosylation reactions by up to 1800-fold [21]. Similarly, neighbouring aromatics have been shown to have an effect on radical reactions proceeding through a polarised transition state [22]. Clearly there is excellent potential, therefore, for aromatic interactions to mediate reactions involving anions [23], and it is likely that these types of interactions will be extremely relevant in catalysis, particularly in biological systems [24].

**Figure 1:**
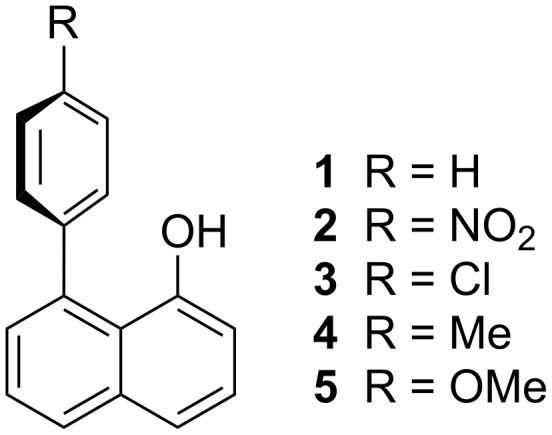
1,8-disubstituted naphthalene model systems.

To probe this possibility in more detail, we have prepared a selection of simple model systems **1**–**5**, based on 1,8-disubstituted naphthalene (Figure 1). Such model systems have already been utilised to great effect by Cozzi and co-workers to probe π–π interactions [25–26]. Related models have also proven effective in exploring neighbouring group interactions in reactive systems, such as phosphate hydrolysis [27–28]. In order to analyse any effects of the aromatic system on the p*K*_a_ value of the naphthols **1**–**5** in a complementary fashion, density functional calculations using the M06-2X functional [29], and atoms in molecules (AIM) [30], analyses have been used.

## Results

**Scheme 1:**
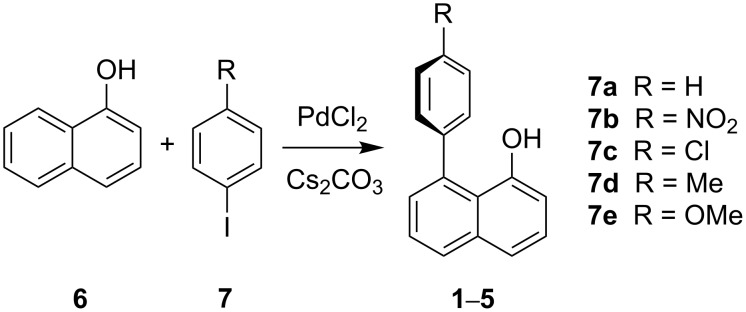
The general reaction for the preparation of the 1,8-disubstituted naphthol derivatives **1**–**5** [31].

Preparation of the 1,8-disubstituted naphthalenes **1**–**5** was carried out following literature procedures [31]. 1-Naphthol (**6**) was reacted with an 1-iodo-4-R-benzene **7** [R = (a) H, (b) NO_2_, (c) Cl, (d) Me, (e) OMe] in the presence of a PdCl_2_ catalyst and Cs_2_CO_3_ in DMF at 110 °C for 19–43 h, under Schlenk conditions (Scheme 1). Reactions were continued until all the starting material was consumed, as determined by TLC. It was noted that iodobenzenes with larger substituents [(d) Me, (b) NO_2_ and (e) OMe] took longer to react, suggesting steric, rather than electronic, limitations in the rate-determining step of the reaction.

**Figure 2:**
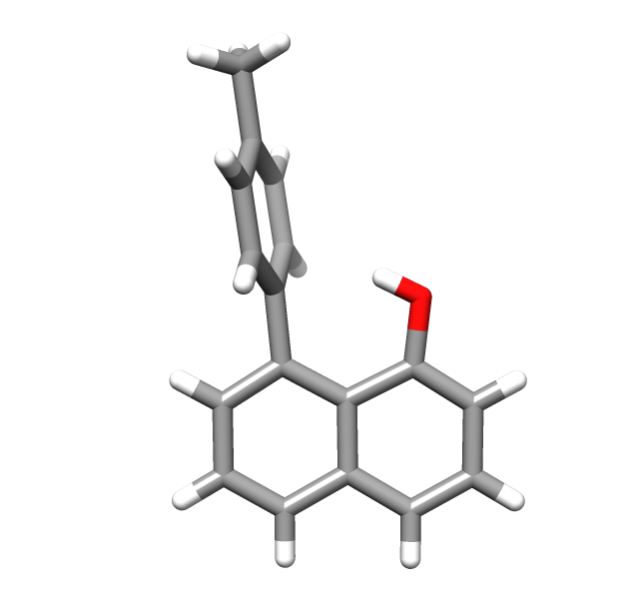
X-ray structure of 8-(4-methylphenyl)-1-naphthol derivative **4**.

The five different substituents were chosen to span the range of electronic effects that could be invoked as a neighbouring effect. The identity of the products **2**–**5** was confirmed through melting point, ^1^H and ^13^C NMR spectral data, IR and MS (Supporting Information File 1). The 8-(4-methylphenyl)-1-naphthol derivative **4** was also crystallised and an X-ray crystal structure obtained, confirming the structure (Figure 2). Details are supplied in the Supporting Information Files 4–6.

**Figure 3:**
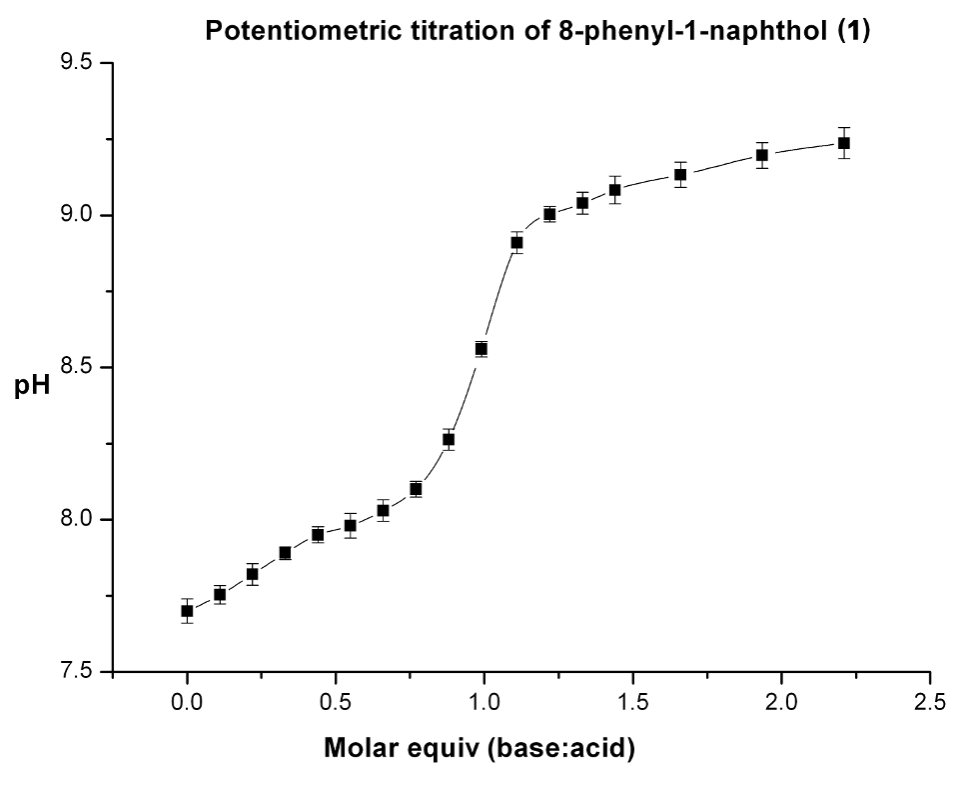
Potentiometric titration data for compound **1** and TBAH.

The acid dissociation constants (*K*_a_'), and hence the p*K*_a_' values of each of the five derivatives **1**–**5**, were measured by potentiometric titration in 50:50 mixtures of acetonitrile/water with tetrabutylammonium hydroxide (TBAH) as the base. As an example, the plot obtained for compound **1** with TBAH is shown in Figure 3.

The derived p*K*_a_' values, along with the Hammett σ_p_-constant for each R-substituent are presented in Table 1. The method was validated with 1-naphthol, for which the experimental p*K*_a_ value is reported as 9.30 [32–33].

**Table 1:** p*K*_a_' Values of 1-naphthol and the derivatives **1**–**5**, along with the corresponding σ_p_ values. Errors calculated on the basis of standard deviations from triplicate measurements.

Derivative	p*K*_a_' Value	σ_p_ Value [34]

**6** (1-Naphthol)	9.31 ± 0.04	n/a
**5** (OMe)	8.71 ± 0.05	−0.29
**4** (Me)	8.68 ± 0.04	−0.17
**1** (H)	8.56 ± 0.03	0.00
**3** (Cl)	8.52 ± 0.05	0.22
**2** (NO_2_)	8.42 ± 0.04	0.77

**Figure 4:**
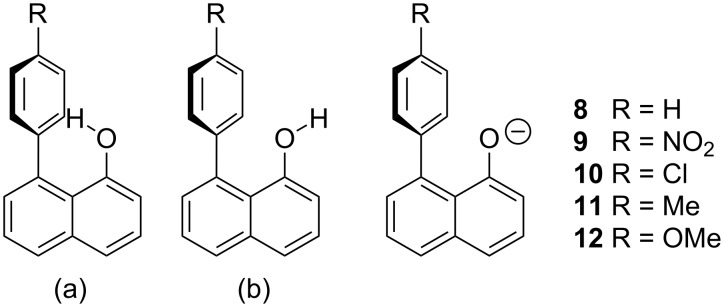
Structures (a) with the hydrogen atom pointing into the ring, as seen in the crystal structure of **4**, and (b) with the hydrogen atom pointing away from the ring and the anions **8**–**12**.

Gas phase calculations for the H, NO_2_ and OMe substituted derivatives **1**–**5**, respectively, and their corresponding anions, **8**–**12**, were carried out to delineate the factors contributing to the experimental p*K*_a_' values. These were carried out using the M06-2X functional [29] with the 6-31+G(d,p) basis set. This parameterised functional has been shown to provide reliable values for intermolecular interactions, including hydrogen-bonding interactions [35]. For the acid, two minima were identified; one with the hydrogen atom pointing into the neighbouring aromatic ring (a) and one with the hydrogen atom pointing away from the ring (b) (Figure 4). The relative energies for each derivative and its corresponding anion are presented in Table 2. Structures are supplied in the Supporting Information File 2.

**Table 2:** Relative energies (kJ·mol^−1^) of protonated species **1**–**5** and their corresponding anions **8–12**, relative to 1-napthol (**6**), calculated with the M06-2X DFT method. Mean standard error (MSE) for mixed systems with M06-2X/6-31+G(d,p) has been reported as −1.0 kJ·mol^−1^ [35].

Species	H-in (a)		H-out (b)^a^	Anion^b^	
	
	6-31+G(d,p)	6-311+G(3df,2p)		6-31+G(d,p)	6-311+G(3df,2p)

**1** (H)	−9.7	−10.2	0	−5.6	−7.5
**2** (NO_2_)	−0.2	−1.6	0	−41.5	−42.6
**3** (Cl)	−6.1	−7.1	0	−19.7	−21.3
**4** (CH_3_)	−11.7	−12.3	0	−4.6	−6.4
**5** (OCH_3_)	−11.5	−12.3	0	−3.8	−5.7

All values included zero point corrections, scaled to 0.967 [36]. ^a^Set arbitrarily at zero for comparison across each row. Either smaller or more negative numbers denote more stable species. ^b^Calculated from the isodesmic reaction with naphthol **6** to afford the corresponding anion, using H-out (b) as the neutral. Larger negative values indicate anions relatively more stable with respect to naphtholate.

## Discussion

Intermolecular effects on molecules are widely recognised as being important in both binding and reactions, with solvent effects being the classic example of the latter. The effects of solvation on reactive intermediates can change the outcome of a reaction, primarily by modifying the rate. In these cases, interactions with the reactive intermediate or with the starting material can serve to accelerate a process, for example, by stabilisation of the intermediate or by activation of the starting material, or decelerate it in an analogous fashion. In restricted model systems and in enzyme active sites, in which the interacting species are brought in close proximity to one another, these effects are often amplified because of the reduction of the contribution of entropic factors. This is recognised as a proximity effect and can be measured by an effective molarity [37]. As such, we chose the 1,8-disubstituted naphthalenes as model systems to examine the effect of a neighbouring aromatic ring on one of the simplest reactions, the removal of a proton from an acid to generate an anion. Cozzi and co-workers examined related models extensively in the study of arene–arene interactions [25–26]. These models were utilised because rotation to generate a conjugated biphenyl system is aggravated by steric interactions [38] and contributions from para-substitution towards generating such a conjugated system are very small [39]. The lack of conjugation is corroborated in the solid state by the X-ray crystal structure of compound **4**, which shows the structure as having the substituted aromatic ring roughly perpendicular to the naphthalene rings. These factors render the structures **1**–**5** suitable for the current study.

**Scheme 2:**
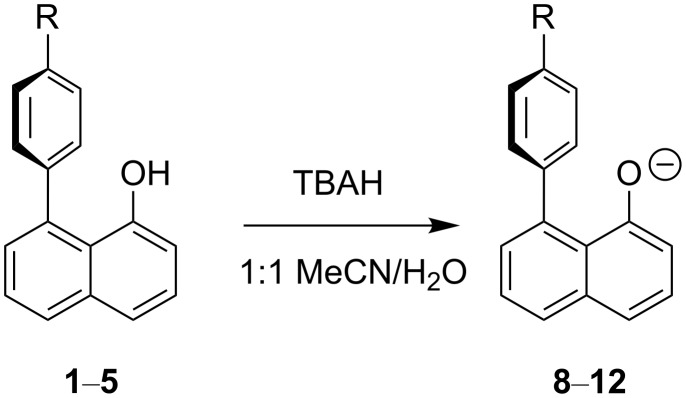
Titration of the acids **1**–**5** to generate the corresponding anions **8**–**12**, respectively.

Compounds **1**–**5** were titrated with TBAH to generate the corresponding anions **8**–**12** (Scheme 2). Potentiometric titrations of acidic compounds are normally conducted by adding aliquots of a base to an acid in an aqueous solution. However, all five derivatives **1**–**5**, as well as naphth-1-ol (**6**) itself, are insoluble in water. A substitute was therefore required with similar properties to water that would enable the calculation of the appropriate relative p*K*_a_ values. There has been a substantial amount of literature produced on potentiometric titrations in binary solvent systems of a 50:50 mix of water and a solvent that dissolves the relevant compound [40–41].

Acetonitrile has been shown to be the most suitable solvent for the p*K*_a_' determination as it has the closest properties to water [42], therefore the titrations were carried out in a 50:50 mix of water and acetonitrile, with the pH meter calibrated against standard calibrants fully dissolved in this solvent mixture. Solvent effects will nevertheless impact upon the p*K*_a_ calculated, therefore p*K*_a_ values derived from compounds in binary solvent systems are represented with a prime symbol (') indicating the p*K*_a_ values are not measured in pure water.

Solvents can affect the properties of acids in three ways; firstly, protic solvents encourage ionisation of the acid via hydrogen bonding. However, acetonitrile is non-protic, which means this does not need to be considered. Secondly, the basicity of the solvent affects the acidity of a compound; the more basic a solvent, the more an acid dissociates. Acetonitrile and water have very similar donor numbers of 14.1 and 18, respectively [43], therefore the p*K*_a_ value of each of the acids **1**–**5** will be barely affected through this effect. The final effect that may influence the p*K*_a_ value of an acid is through homoconjugation, where the conjugate base hydrogen bonds to the parent acid. This does not occur to a significant extent in water, as the water forms strong hydrogen bonds with itself. The extent of homoconjugation that may occur can be gauged by the dielectric constant (ε) of the solvent, where a lower dielectric constant corresponds to a higher extent of homoconjugation, which increases the acidity and lowers the p*K*_a_ value of an acid. Acetonitrile and water have dielectric constants of 36 and 81.7 [42,44], respectively. As a consequence of the difference in the dielectric constants, there may be a slight difference in the measured p*K*_a_' value and the actual p*K*_a_. It is worth remembering, however, that the resulting p*K*_a_' value of every derivative is relative to the others and therefore any effects of the R-substituents are likely to be preserved.

**Figure 5:**
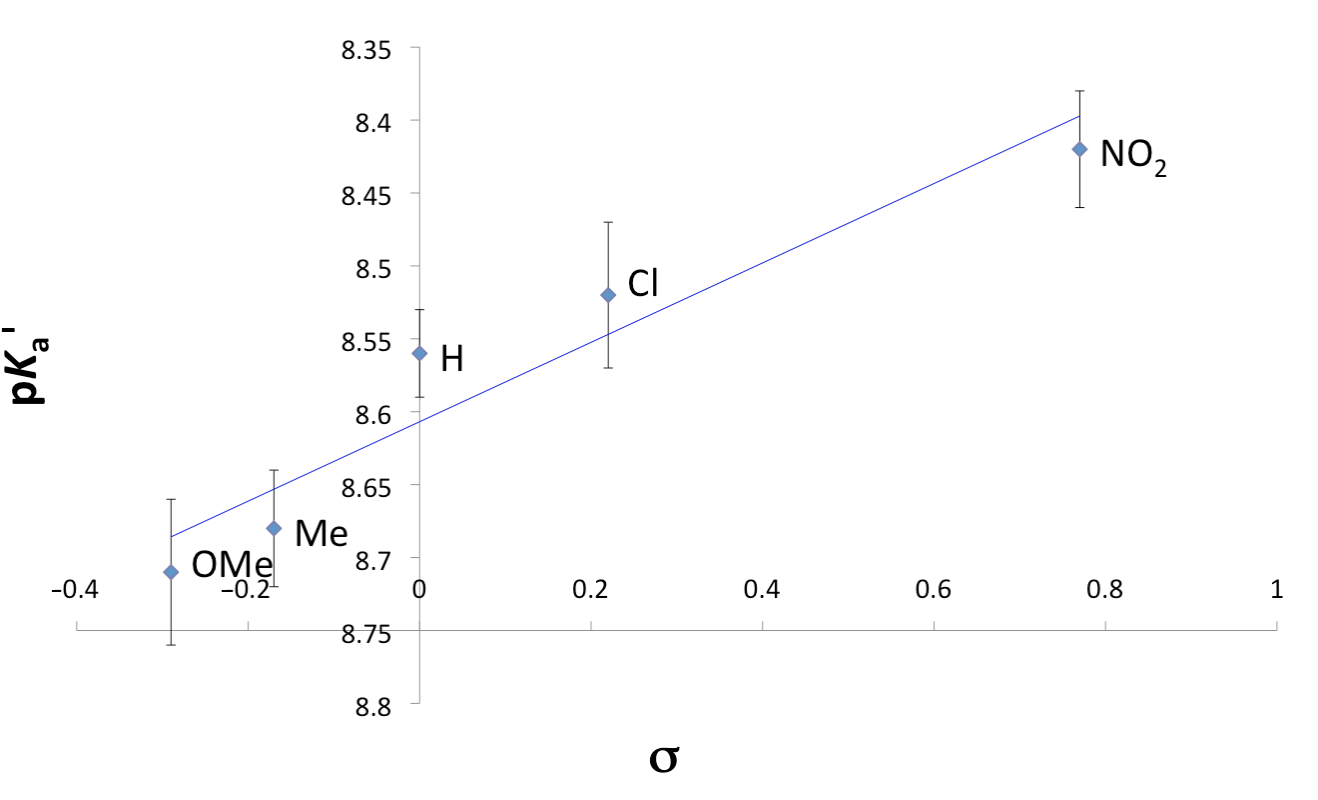
Plot of p*K*_a_' values for compounds **1**–**5** versus the corresponding R-substituent σ_p_ Hammett parameter. The linear correlation has R^2^ of 0.916.

The p*K*_a_' values correlate linearly with the corresponding Hammett σ_p_-constants for the R-substituents, affording a correlation constant (R^2^) of 0.916. The linear dependence is illustrated in Figure 5. The slope of the Hammett plot confirms that the intermediate or product is stabilised by electron deficient groups, consistent with deprotonation. The magnitude of the slope is relatively small (−0.27), as might be expected for a through-space effect, and may also be indicative of solvation reducing the apparent localised charge.

For a better interpretation of the factors contributing to the changes in measured p*K*_a_' values, M06-2X density functional calculations of both the starting materials **1**–**5** and the corresponding anions **8**–**12** were carried out. Such calculations generate both energetic information and structural information, which may be otherwise difficult to obtain for the anion intermediates. These calculations indicate that the nature of the differences in p*K*_a_' values are two-fold. In the first instance, there is a small contribution from differences in binding of the naphtholic hydrogen atom to the neighbouring ring (Figure 4a), measured relative to the alternative minimum-energy orientation (Figure 4b). This interaction is tightest for the electron-rich ring of the methoxy-substituted acid **5**, and would result in more difficult abstraction of the proton, relative to less electron-rich acids, such as **2**. In fact, for the acid **2**, the difference is negligible, suggesting that this could be a simple electrostatic interaction. The nature of this interaction was confirmed by AIM analysis of **1**(a)–**5**(a) and the ρ values for relevant critical points, for these molecules and anions **8**–**12**, are included in Table 3. For these derivatives, a non-covalent bonding interaction is detected as a bond critical point between the naphthol proton and the quaternary (1') carbon of the 8-substituent and is accompanied by a ring critical point from the 6-membered ring made from the additional atoms of the naphthalene moiety. The ρ value is, as can be surmised from the lower interaction energy, lower for the bond critical point between H and C for **2**(a) (0.0193 e·bohr^−3^) than for that between H and C for **1**(a) (0.0223 e·bohr^−3^). Molecular graphs are supplied in the Supporting Information File 3.

**Table 3:** Electronic densitites (ρ) (e·bohr^−3^) and Laplacian (Lp) values 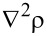 (e·bohr^−5^) of the identified bond critical points for protonated (OH-C1') and deprotonated (O-C1') species **1**–**5** and their corresponding anions **8–12**, as determined from AIM analysis.

Species	H-in (a)		Anion	

	ρ	Lp ρ	ρ	Lp ρ

**1** (H)	0.0223	−0.0175	0.0163	−0.0155
**2** (NO_2_)	0.0193	−0.0162	0.0176	−0.0167
**3** (Cl)	0.0215	−0.0171	0.0167	−0.0162
**4** (CH_3_)	0.0227	−0.0176	0.0162	−0.0155
**5** (OCH_3_)	0.0230	−0.0176	0.0164	−0.0158

The second component that is likely to contribute to the measured p*K*_a_' values is the interaction between the anion and the neighbouring aromatic ring. Because of the difference in the system from the starting materials, namely one less bond and thus calculation through an isodesmic procedure, the values are best compared within the series. This interaction is again least strong for the nitro derivative **2**, indicating either a less unfavourable interaction [18] or a more favourable one. In addition, this latter effect seems to reach saturation with electron-rich rings, consistent with the argument that the differences in anion–π interaction are primarily a result of quadrupole interactions.

The two effects observed act together, and are thus consistent with the trend observed for the p*K*_a_' values. It is worth noting that, relative to gas-phase calculations, the measured experimental effect is likely to be attenuated by hydrogen-bonding interactions with solvent. Such an interaction with solvent may not, however, be relevant in some enzyme and supramolecular systems.

**Figure 6:**
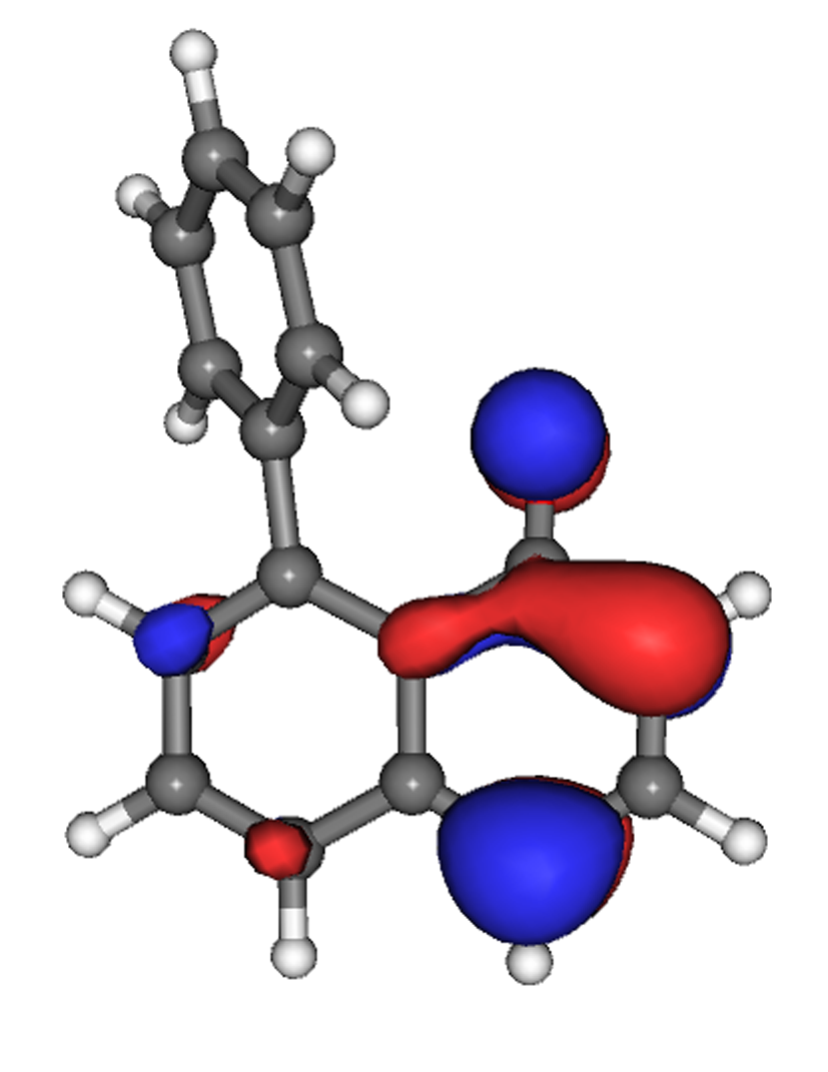
Anion density (HOMO) for the phenyl-derivative **8**, illustrating no conjugation of the anion with the neighbouring aromatic.

The minimised structures for each of the anions illustrate an interesting feature. There is consistently an increased twist from the perpendicular plane of the naphthalene, such that the angle is around 130°, rather than the ca*.* 120° of the starting material. This, however, is not enough to bring the substituted ring into conjugation with the anion, as indicated by the distribution of the HOMO, which is confined to the naphthol portion of each molecule, and is in fact principally located on the phenolic ring (illustrated for **8** in Figure 6). This may, however, bring the oxyanion closer to the more positively charged periphery of the neighbouring aromatic ring.

**Figure 7:**
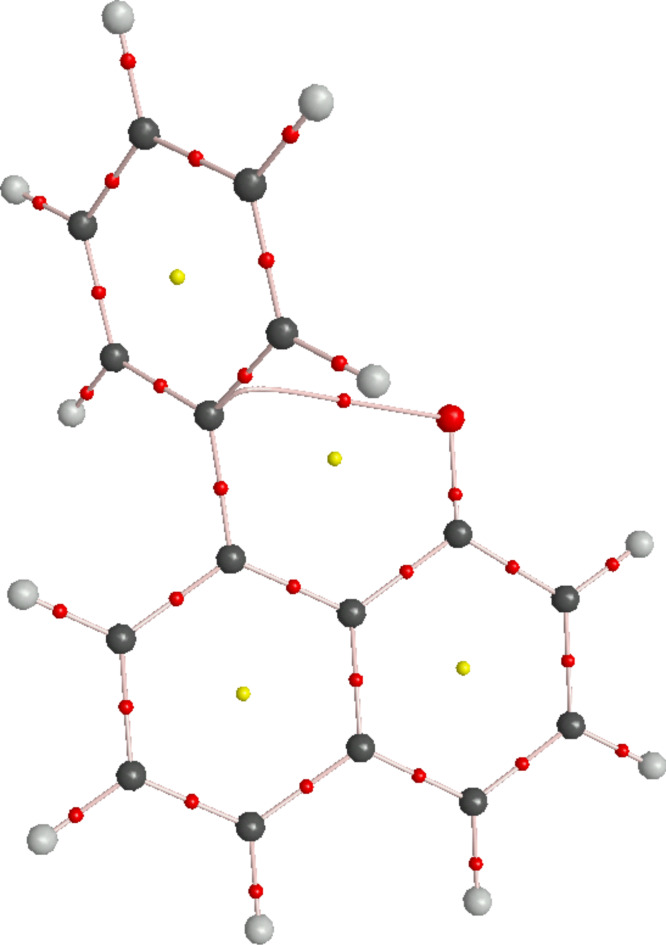
Bond critical points (red), ring critical points (yellow) and bond paths illustrated for the anion **8**, generated by AIM2000.

AIM analysis of the anions revealed a bond critical point between the oxygen and the C1' carbon (Figure 7) with positive ρ of 0.0163. This indicates that the interaction between the ring and the anion is not just one of proximity, but does indeed constitute a ‘bond’ that can, in principle, be classified as a true anion–π interaction.

## Conclusion

The effects of proximal aromatic residues on anions have been described in the literature extensively in the context of binding studies. We have examined one of the simplest reactions, proton abstraction, with the five 8-(4-R-phenyl)-1-naphthol derivatives **1**–**5**. These derivatives exhibit changes in their p*K*_a_' values consistent with the changing electronic nature of R substituent, suggesting an anion–π effect in modulating the hydrogen abstraction process. Density functional calculations indicate that the change in reactivity is likely to be dependent on two factors: a stronger OH-C1' interaction with more electron-rich character, making the hydrogen more difficult to abstract, and an increased stability of the anion with electron-poor substituents, relative to electron-rich aromatics. AIM analysis revealed bond critical points that suggest that the OH-C1' interaction can indeed be classified as a bond, as well as an anion–π interaction between the anion oxygen and C1'. Such interactions are likely to have an impact on related reactions where anions are formed in close proximity to an aromatic ring and indicate that anion–π interactions could be used in supramolecular architectures to modulate reactivity. Likewise, interactions of this type may play a guiding role in some enzyme reactions.

## Experimental

### General

The ^1^H and ^13^C NMR spectra were recorded on a Bruker Avance 500 Digital NMR spectrometer at 500 MHz in CDCl_3_. GC-MS spectra were recorded on an Agilent Technologies 6890N network GC system. All IR spectra were recorded on a Perkin Elmer 100 FT-IR spectrometer. The UV–vis spectra were recorded on a Unicam UV–vis spectrometer UV 4.

Pure 1-naphthol **6** was required to increase the yield of the desired product. Once exposed to air, over time, 1-naphthol **6** degrades to form impurities and the crystals change colour from white to dark grey. Crystals that were not white were purified by the following method [45]: 1-Naphthol (**6**) (3–4 g) was placed in the bottom of a purpose-built sublimator. Water pumps were attached to the sublimator and chilled water was run through the system. The system was then connected to a vacuum. The solid was then heated in an oil bath to 90 °C (just below the melting point of 1-naphthol of 95.5–96.0 °C); care was taken not to heat the oil bath above the melting point of naphthol. After 2 h the solid was removed from the heat and left to cool. Pale yellow crystals had formed on the cold finger. These crystals were placed in a round-bottomed flask (50 cm^3^) and dissolved in hot 25% aq ethanol (5 cm^3^). After the crystals had dissolved, they were left to cool in an ice bath and then filtered using a Buchner funnel. The crystals were washed with deionised water and dried for 24 h in a vacuum desiccator with P_2_O_5_ as the drying agent.

The DMF used for the reactions must be dry, as water must not enter the system while the reaction is taking place. Dry DMF was prepared by the following method:

DMF was run over silica gel and the run off collected in a round-bottomed flask. The flask was placed on a rotary evaporator and 10% of the liquid evaporated to remove any low boiling impurities (DMF has a high boiling point of 153 °C so any low boiling material can be considered as unwanted impurities). The dry DMF was stored over molecular sieves (3 Å) in a dark bottle below 5 °C. The DMF stayed dry for three weeks under these conditions. Before using the DMF the bottle was removed from the fridge and left to warm up to room temperature. If the bottle was opened whilst cold, condensation formed on the walls of the bottle and contaminated the DMF.

All other chemicals were used as supplied, with ^1^H NMR spectra and either the melting points or boiling points of all materials recorded to confirm identity and purity.

### Arylation of 1-naphthol

Cs_2_CO_3_ (10 mmol) was placed in a two-necked round-bottomed flask (100 cm^3^) and dried in vacuo (150 °C, 2 h). PdCl_2_ (0.125 mmol), 1-iodo-4-R-benzene **7** (6 mmol), naphthol **6** (5 mmol) and DMF (25 cm^3^) were added to the pre-dried base. Upon addition of the reactants, the mixture turned dark green/black. The reaction mixture was stirred under a nitrogen atmosphere and heated (110 °C, 19–43 h, Table 4) then left to cool and extracted twice with diethyl ether (25 cm^3^) and water (25 cm^3^), and once with brine (25 cm^3^). The extracts were dried with magnesium sulfate. The products (**1**–**5**) were isolated by column chromatography on silica gel with hexane/ethyl acetate as the eluent.

**Table 4:** Time taken for reaction to occur for each of the five derivaties of **1**–**5**.

Product	Reaction time (h)	Isolated yield (%)

**1**	19	81
**4**	39	45
**3**	21	68
**5**	43	46
**2**	42	77

**8**-**phenyl**-**1**-**naphthol (1):** Oil; ^1^H NMR δ = 5.29 (s, 1H), 6.78 (d, 1H, *J* = 6.3 Hz), 7.05 (d, 1H, *J* = 6.9 Hz), 7.24 (t, 1H, *J* = 7.9 Hz), 7.28 (t, 1H, *J* = 8.2 Hz), 7.28–7.36 (m, 6H), 7.71 (d, 1H, *J* = 6.5 Hz) ppm; ^13^C NMR δ = 111.9, 121.3, 125.0, 127.0, 128.7, 129.1, 135.9, 141.5, 153.2 ppm.

**8**-**(4**-**nitrophenyl)**-**1**-**naphthol (2):** mp 135–135.5 °C; ^1^H NMR δ = 7.02 (d, 2H, *J* = 9.15 Hz), 7.17 (s, 1H), 7.50 (m, 3H), 7.79 (d, 1H, *J* = 8.5 Hz), 7.93 (d, 2H, *J* = 9.15 Hz), 8.20 (d, 2H, *J* = 9.45 Hz) ppm; ^13^C NMR δ = 116.6, 121.6, 125.8, 125.9, 126.1, 126.7, 126.9, 127.0, 128.2, 135.2, 150.4 ppm; IR (nujol mull) ν: 2000–1650, 1592, 1507, 1488, 1342, 1244 cm^−1^; MS *m*/*z* 265 (M^+^).

**8**-**(4**-**chlorophenyl)**-**1**-**naphthol (3):** mp 44–46 °C; ^1^H NMR δ = 5.20 (s, 1H), 6.92 (dd, 1H, *J* = 0.95, 7.55 Hz), 7.18 (dd, 1H, *J* = 0.95, 6.95 Hz), 7.39–7.46 (m, 7H), 7.86 (dd, 1H, *J* = 0.95, 8.2 Hz) ppm; ^13^C NMR δ = 112.1, 121.4, 125.1, 127.0, 128.9, 130.9, 134.7, 135.9, 140.2, 152.8 ppm; IR (nujol mull) ν: 3548, 2000–1650, 1526, 1488, 1457 cm^−1^; MS *m*/*z* 254 (M^+^).

**8**-**(4-methylphenyl)**-**1**-**naphthol (4):** mp 77–79 °C; ^1^H NMR δ = 2.46 (s, 3H), 5.56 (s, 1H), 6.91 (dd, 1H, *J* = 1.25, 6.3 Hz), 7.18 (dd, 1H, *J* = 1.25, 6.9 Hz), 7.41 (d, 2H, *J* = 7.85 Hz), 7.42–7.49 (m, 5H), 7.85 (dd, 1H, *J* = 1.25, 7.25 Hz) ppm; ^13^C NMR δ = 21.4, 111.8, 121.4, 125.0, 127.0, 128.6, 136.3, 138.3, 138.8, 153.3 ppm; IR (nujol mull) ν: 3532, 1582, 1262, 822; MS *m*/*z* 234 (M^+^).

**8**-**(4-methoxyphenyl)**-**1**-**naphthol (5):** mp 167–168 °C; ^1^H NMR δ = 1.60 (s, 3H), 3.90 (s, 1H), 5.26 (dd, 1H, *J* = 0.95, 8.85 Hz), 6.81–7.33 (m, 7H), 7.81 (dd, 1H, *J* = 0.95, 9.45 Hz), 8.18 (dd, 1H, *J* = 0.95, 9.15 Hz) ppm; ^13^C NMR δ = 55.5, 108.7, 111.8, 114.6, 120.8, 121.7, 124.5, 125.4, 127.8, 134.9 ppm; IR (nujol mull) ν: 3516, 2000–1650, 1539, 1464, 1377 cm^−1^; MS *m*/*z* 250 (M^+^).

### Measurement of p*K*_a_ values

The p*K*_a_ values for the derivatives **1**–**5** dissolved in 50:50 water/acetonitrile solution were determined by potentiometric titration using a PHM210 Standard lab pH meter that had been calibrated against standards (pH 4 and pH 7). The electrode was first placed in 50 ml of a known concentration of an acid **1**–**5** and the solution was constantly stirred to ensure equilibration. To this sample of **1**–**5**, 20 μL aliquots of TBAH (0.034 mol·L^−1^, 50:50 water/acetonitrile) were added. The solution was allowed to equilibrate for 30 s after each addition, and the pH was recorded. This process was repeated until three millilitres of base had been added, which corresponded to a molar ratio of base to acid of approximately 3:1, depending on the acid. This ratio was more than sufficient to fully deprotonate the acid as the half way neutralisation point (HNP) occurs when the ratio is 1:1. This process was carried out in triplicate for all of the derivatives and the results were averaged out over all three titrations.

The action of TBAH was confirmed through UV–vis spectroscopy. First the molar absorption coefficient (ε) for each acid **1**–**5** was determined by producing standard curves from known acid concentrations ranging from 4 × 10^−4^ to 1 × 10^−4^ by applying the Beer–Lambert law. The UV–vis spectra of all five derivatives of **1**–**5** showed a shift in the λ_max_ value upon addition of TBAH. This shift and the corresponding molar absorption coefficients are presented in Table 5.

**Table 5:** Table indicating the standard concentrations to use for the titrations along with the relevant UV–vis spectral data.

Derivative	Concentration (× 10^−4^ mol·L^−1^)	λ_max_ of acid (nm)	ε of acid (mol·L^−1^·cm^−1^)	λ_max_ of deprotonated acid (nm)	ε of deprotonated acid (mol·L^−1^·cm^−1^)

**6**	3	296	7640	332	38660
**1**	2.5	308	6753	332	4265
**2**	1.5	292	34240	304	2069
**3**	2	348	1298	308	19790
**4**	2	316	6242	304	10720
**5**	4	292	2324	296	3595

### Calculation of 1,8-disubstituted naphthols 1–5 and their corresponding anions **8**–**12**

DFT calculations were performed using the Gaussian 09 package [46]. The parameterised functional M06-2X with the 6-31+G(d,p) basis set was used for geometry optimisations. All optimised structures are local minima, as confirmed by frequency calculations. The minimum energy conformers of all molecules examined have *C*_1_ symmetry. Vibrational frequencies and zero point energies were calculated by the M06-2X method, and scaled by 0.9670 [36]. AIM analyses were performed using XAim on Gaussian wfn output to examine densities and laplacians [47], and AIM2000 to identify critical points [48]. To generate the wfn files, M06-2X/6-311+G(3df,2p) single point energies were calculated.

## Supporting Information

File 1 Structural data for compounds **1**–**6** and **8**–**12** optimised at M06-2X/6-31+G(d,p).

File 2 Original spectral data for compounds **1**–**5**.

File 3 Molecular graphs for molecules **1**(a)-**5**(a) and **8**-**12**.

File 4 Crystal information file for compound **4**.

File 5 Crystal information data file for compound **4**.

File 6 Crystal structure refinement details for compound **4**.
