# Supplementary material for: Anion–π interactions influence pKa values
Source: Beilstein J Org Chem. 2011 Mar 17;7:320–8. doi: 10.3762/bjoc.7.42 (PMC3079108; doi:10.3762/bjoc.7.42)
Supplement: File 1 — Structural data for compounds 1–6 and 8–12 optimised at M06-2X/6-31+G(d,p). [file Beilstein_J_Org_Chem-07-320-s001.doc]

Supporting Information

for

Anion–π interactions influence p*K*_a_ values

Christopher J. Cadman and Anna K. Croft*^*^*

Address: School of Chemistry, University of Wales Bangor, Bangor, Gwynedd, LL57 2UW, United Kingdom. Fax: +44 1248 370 528. Tel: +44 1248 382 375.

Email: Anna K. Croft - [*a.k.croft@bangor.ac.uk*](mailto:a.k.croft@bangor.ac.uk)

^*^Corresponding author

**Structural data for compounds 1**–**6 and 8**–**12 optimised at M06-2X/6-31+G(d,p)**

**1(a)**

1\1\GINC-ABE0599\FOpt\RM062X\6-31+G(d,p)\C16H12O1\ACROFT\24-Jul-2010\0

\\#p M062X/6-31+G(d,p) opt freq=noraman\\1-phenyl-8-napthol H-in with

twist\\0,1\C,0.0343590963,0.2153906098,-0.0621611669\C,-0.0501061228,0

.1704869111,1.3270905317\C,1.0937769687,-0.0603354361,2.0911654145\C,2

.3218042321,-0.2460184758,1.4593895437\C,2.4077420425,-0.2012652315,0.

0686712246\C,1.2662190986,0.0340726965,-0.707752015\C,1.342659532,0.01

52173713,-2.1999551284\C,1.0305708078,1.1452791647,-3.0317046759\C,0.7

913762717,2.4804898073,-2.5561615834\C,0.4485480059,3.4892345797,-3.43

14900297\C,0.3605308096,3.2536112724,-4.8153459927\C,0.6375879825,2.00

93117247,-5.3213349813\C,0.9846892075,0.9459222417,-4.4497766012\C,1.3

083138486,-0.3281382667,-4.9905840743\C,1.6672062504,-1.3705401018,-4.

1799345287\C,1.6717444341,-1.1923637407,-2.7788744542\H,1.8992390778,-

2.0335748917,-2.1302166161\H,1.9225968895,-2.3375133071,-4.6013559263\

H,1.2712708188,-0.4521510432,-6.0694129182\H,0.6029059494,1.8183156637

,-6.3895772825\H,0.0930195459,4.0703818002,-5.4782269219\H,0.273645046

3,4.4762030274,-3.0169433098\O,0.9051016739,2.8590635742,-1.2565411761

\H,1.1803564174,2.130397394,-0.6876593928\H,3.3647067318,-0.339194675,

-0.4270615447\H,3.2164258588,-0.4228491638,2.0482759476\H,1.0277802196

,-0.0930622668,3.1740426785\H,-1.0106860609,0.313700389,1.811856298\H,

-0.8547865553,0.3955728681,-0.660659667\\Version=EM64L-G09RevA.02\Stat

e=1-A\HF=-691.9092171\RMSD=8.212e-09\RMSF=5.473e-06\Dipole=0.1118314,-

0.6721253,0.313443\Quadrupole=-3.1761944,-3.9460234,7.1222178,-2.79789

02,0.8052998,-1.7873758\PG=C01 [X(C16H12O1)]\\@

**1(b)**

1\1\GINC-ABE0172\FOpt\RM062X\6-31+G(d,p)\C16H12O1\ACROFT\23-Jul-2010\0

\\#p M062X/6-31+G(d,p) opt freq=noraman\\1-phenyl-8-napthol H-out with

twist\\0,1\C,0.0425256016,0.2309716126,-0.0376248035\C,-0.0251235641,

0.1724794493,1.349929795\C,1.1265822414,-0.0757629812,2.0987482602\C,2

.3416588211,-0.2714288552,1.448101163\C,2.4061945931,-0.2192733634,0.0

556209853\C,1.260495507,0.0409770029,-0.6988630016\C,1.3180731572,0.01

37105779,-2.1907605185\C,1.015660573,1.1344927997,-3.0345368094\C,0.79

16204269,2.4672895917,-2.5562253583\C,0.4741769358,3.4905817018,-3.420

9321925\C,0.3895359728,3.2634012846,-4.811038448\C,0.6424556787,2.0172

783497,-5.318361732\C,0.9695704439,0.9402762649,-4.4500935196\C,1.2726

636578,-0.3366866828,-4.991435685\C,1.6164361841,-1.3801747824,-4.1738

097855\C,1.6283209933,-1.1987724578,-2.7738497015\H,1.8517775539,-2.04

12499921,-2.1257490697\H,1.8577574828,-2.3523514397,-4.5921130051\H,1.

2341318945,-0.4638038911,-6.0697704786\H,0.6044475463,1.831917044,-6.3

87508915\H,0.1391842391,4.0882669137,-5.4703239297\H,0.3051405316,4.48

84148206,-3.0230098673\O,0.9315791655,2.692395451,-1.2225232677\H,0.80

34084053,3.6300697661,-1.0383396689\H,3.3546682521,-0.3683833831,-0.45

34141906\H,3.2427656544,-0.4659540122,2.0218652717\H,1.0740276486,-0.1

194044927,3.1822954887\H,-0.9781935764,0.3184355855,1.849599098\H,-0.8

523177646,0.4300200645,-0.6213581661\\Version=EM64L-G09RevA.02\State=1

-A\HF=-691.9052856\RMSD=3.879e-09\RMSF=3.071e-06\Dipole=-0.1025498,0.5

598395,-0.1119371\Quadrupole=-5.2274829,3.2088747,2.0186081,-3.8067641

,0.1133103,1.4538333\PG=C01 [X(C16H12O1)]\\@

**2(a)**

1\1\GINC-ABE0552\FOpt\RM062X\6-31+G(d,p)\C16H11N1O3\ACROFT\01-Aug-2010

\0\\#p M062X/6-31+G(d,p) opt freq=noraman int=ultrafine\\1-nitrophenyl

-8-napthol hin twist M06-2X/6-31+G(d,p) opt//\\0,1\C,0.1089966972,0.34

61962701,-0.0670704214\C,0.0142822234,0.3083204411,1.3181591941\C,1.14

67633732,-0.0321173402,2.0492131322\C,2.3618226783,-0.3316776574,1.448

2946621\C,2.4394994568,-0.2896058544,0.0597603192\C,1.3195223642,0.048

0240694,-0.7136619148\C,1.3881308022,0.0064698709,-2.2029397713\C,1.02

61501948,1.1083118566,-3.0531892063\C,0.7974069907,2.4543728969,-2.610

2078441\C,0.3755458893,3.4256043141,-3.4922570009\C,0.2094036914,3.141

1045123,-4.8600237683\C,0.4981717836,1.8901827393,-5.3431513318\C,0.92

04068752,0.8643570415,-4.4605136966\C,1.2589624371,-0.415493479,-4.978

8538218\C,1.6826759744,-1.4233001808,-4.1560366714\C,1.7301494858,-1.2

080687244,-2.7611019058\H,1.9889372924,-2.0314258205,-2.1013935707\H,1

.9494283037,-2.3941934108,-4.5600479289\H,1.1803186781,-0.5717724429,-

6.0510324351\H,0.416528282,1.6659325026,-6.4021478584\H,-0.1185898884,

3.9283884988,-5.5309581442\H,0.2087484799,4.4233237066,-3.1011916656\O

,0.9884326072,2.8750931762,-1.3302837948\H,1.4387252342,2.2127949043,-

0.7950010475\H,3.3795170558,-0.5111769017,-0.4361072688\H,3.2177287953

,-0.5857779887,2.0617645635\N,1.0540576924,-0.0765891787,3.5195287232\

O,2.0591742375,-0.3781329149,4.1378966697\O,-0.0219081476,0.1895952885

,4.0236502926\H,-0.9120983885,0.5314692187,1.8332719784\H,-0.760518538

1,0.6086789112,-0.6620217625\\Version=EM64L-G09RevA.02\State=1-A\HF=-8

96.34153\RMSD=6.720e-09\RMSF=7.976e-06\Dipole=0.2532781,-0.4707799,-1.

9587653\Quadrupole=4.8333811,6.2754889,-11.1088701,-3.1304861,1.880157

9,2.5636557\PG=C01 [X(C16H11N1O3)]\\@

**2(b)**

1\1\GINC-ABE0543\FOpt\RM062X\6-31+G(d,p)\C16H11N1O3\ACROFT\01-Aug-2010

\0\\#p M062X/6-31+G(d,p) opt freq=noraman int=ultrafine guess=check ge

om=check\\1-nitrophenyl-8-napthol hout (twist) M06-2X/6-31+G(d,p) opt/

/\\0,1\C,0.0774885464,0.2804715997,-0.0269985828\C,0.0164503643,0.2254

509957,1.3572867039\C,1.1856392962,-0.0491828811,2.0604898081\C,2.3993

034238,-0.2763588434,1.427428606\C,2.4385140193,-0.2226162034,0.037346

362\C,1.2864877163,0.0637343021,-0.7012357646\C,1.3262128073,0.0347821

787,-2.1919426238\C,0.9973354894,1.1517138226,-3.0305130622\C,0.772969

501,2.4830686807,-2.5514410261\C,0.4338397035,3.5017943486,-3.41244553

87\C,0.327053929,3.2696301361,-4.8002639335\C,0.5804712524,2.024010255

5,-5.3090717369\C,0.9296144404,0.9521488158,-4.443964744\C,1.234736671

6,-0.3246096428,-4.9861925312\C,1.6027397288,-1.3628638178,-4.17314437

87\C,1.6394065276,-1.177128652,-2.7738504928\H,1.8817898956,-2.0168375

256,-2.1288944425\H,1.8455705451,-2.3337956262,-4.5924209373\H,1.17912

98478,-0.4548491211,-6.0632170096\H,0.5275240431,1.8360115766,-6.37689

07101\H,0.0597975174,4.0910204554,-5.4569421636\H,0.2650175107,4.49955

28106,-3.0149328512\O,0.9348643045,2.708767468,-1.2190323686\H,0.80984

23284,3.6463105798,-1.0306449906\H,3.3766069365,-0.3898013181,-0.48268

56918\H,3.2832307685,-0.4871667102,2.0169830144\N,1.1316761702,-0.1044

796577,3.5296205905\O,2.1651925524,-0.3540574477,4.1257235414\O,0.0562

549966,0.1037751673,4.0645624602\H,-0.9090818963,0.3868595759,1.896314

085\H,-0.8185710087,0.4982833694,-0.599863426\\Version=EM64L-G09RevA.0

2\State=1-A\HF=-896.3414936\RMSD=3.831e-09\RMSF=3.695e-06\Dipole=-0.05

10488,0.7137912,-2.3679649\Quadrupole=2.301769,13.5193655,-15.8211344,

-3.869858,0.7108829,3.6092405\PG=C01 [X(C16H11N1O3)]\\@

**3(a)**

1\1\GINC-ABE0565\FOpt\RM062X\6-31+G(d,p)\C16H11Cl1O1\ACROFT\29-Jan-201

1\0\\#P M062X/6-31+G(d,p) OPT(EstmFC) FREQ=noraman int=ultrafine outpu

t=wfn\\chloronaphthol OH in\\0,1\C,0.0474301702,0.0826687483,-0.093422

9399\C,-0.0196364522,0.1647354876,1.2935174823\C,1.1590698942,0.108055

184,2.0341580143\C,2.4015437963,-0.0330643258,1.4040088295\C,2.4384021

452,-0.1108957673,0.003971546\C,1.269170045,-0.0559083585,-0.747604771

2\H,1.3008287094,-0.1158468284,-1.8298287219\H,3.3962078796,-0.2179677

149,-0.4975387982\C,3.6671492605,-0.0136539949,2.1961321587\C,4.626717

7018,-1.0846468267,2.1976035934\C,4.4148095839,-2.3860192571,1.6266794

962\C,5.4232247758,-3.3259827943,1.6204039348\C,6.6709930517,-3.057724

7257,2.212137069\C,6.8949094026,-1.8550465384,2.8323428456\C,5.8839125

98,-0.8609966753,2.8471810832\C,6.113596129,0.3646369755,3.5299827786\

C,5.1538785036,1.3386612537,3.5775598145\C,3.9334084613,1.1463173051,2

.8931748209\H,3.1986996553,1.9462452767,2.8712674953\H,5.3326240276,2.

2675336405,4.1092057329\H,7.0718202304,0.5057625717,4.0221567465\H,7.8

393953658,-1.6456044721,3.3249332851\H,7.4427708802,-3.820549454,2.194

8384059\H,5.210626862,-4.2889820274,1.169118302\O,3.2368554869,-2.7978

100138,1.0878561695\H,2.5439845792,-2.1342385232,1.1827967327\H,1.1171

053224,0.1647726158,3.1180173102\H,-0.9810388376,0.26770047,1.78457086

64\Cl,-1.420432486,0.1522779111,-1.0285145528\\Version=EM64L-G09RevA.0

2\State=1-A\HF=-1151.4773523\RMSD=7.008e-09\RMSF=9.600e-06\Dipole=0.32

52464,0.5787528,0.4488832\Quadrupole=1.4567148,-1.6202798,0.163565,-1.

0427539,-1.0945518,2.9083154\PG=C01 [X(C16H11Cl1O1)]\\@

**3(b)**

1\1\GINC-ABE0562\FOpt\RM062X\6-31+G(d,p)\C16H11Cl1O1\ACROFT\29-Jan-201

1\0\\#P M062X/6-31+G(d,p) OPT(EstmFC) FREQ=noraman int=ultrafine\\meth

ylnaphthol OH out\\0,1\C,-0.0005046829,-0.0724220378,0.0459510559\C,0.

0443285642,-0.1217645298,1.4387742903\C,1.2780151515,-0.0739523507,2.0

748026137\C,2.4613706818,0.020293346,1.3457201058\C,2.3980162242,0.075

0778716,-0.0410214096\C,1.1691140499,0.0351836457,-0.7081378433\C,1.11

15193247,0.0060886561,-2.1989426475\C,1.5768482443,1.0662012589,-3.047

6414819\C,1.9949985703,2.3544287376,-2.5780246296\C,2.4586190915,3.314

3027423,-3.449166195\C,2.5080142414,3.0685300209,-4.8378144931\C,2.074

0749597,1.8699896988,-5.3371904498\C,1.593017635,0.8585717474,-4.46211

99943\C,1.1047476816,-0.3630957833,-4.9965833072\C,0.6118744642,-1.340

4353801,-4.1740699454\C,0.6270304871,-1.1513306541,-2.7750408822\H,0.2

834161779,-1.9493108753,-2.1231859455\H,0.2305400785,-2.2689729265,-4.

5866774288\H,1.1232270704,-0.4994723199,-6.0741871181\H,2.0825124773,1

.6742938081,-6.4049852969\H,2.8765233166,3.8436182554,-5.5018728057\H,

2.7728762621,4.2788463365,-3.0577834832\O,1.8924189757,2.6064543076,-1

.2448987139\H,2.1582232429,3.5161581878,-1.0669288726\H,3.3146640577,0

.1553464464,-0.6182819407\H,3.4145888268,0.0475085515,1.8623277454\Cl,

1.349993228,-0.1378518273,3.8186812196\H,-0.8659745372,-0.1958436801,2

.0236064869\H,-0.9607149721,-0.1037472849,-0.4608243101\\Version=EM64L

-G09RevA.02\State=1-A\HF=-1151.4749061\RMSD=4.619e-09\RMSF=3.889e-06\D

ipole=0.1641558,0.5934537,-0.9762813\Quadrupole=-0.315293,5.1793172,-4

.8640243,5.2299622,-0.2032905,1.8763949\PG=C01 [X(C16H11Cl1O1)]\\@

**4(a)**

1\1\GINC-ABE0575\FOpt\RM062X\6-31+G(d,p)\C17H14O1\ACROFT\02-Feb-2011\0

\\#P M062X/6-31+G(d,p) OPT(EstmFC) FREQ=noraman int=ultrafine guess=re

ad geom=check\\methylnaphthol OH in\\0,1\C,0.1181102826,-0.0707314687,

-0.0011334725\C,0.0312687602,-0.0517127611,1.5035698606\C,1.172668336,

0.1833718999,2.2797697559\C,1.1020197194,0.223837442,3.6677887518\C,-0

.1215289739,0.032282649,4.3280784089\C,-1.2619154871,-0.2098673324,3.5

551001286\C,-1.183337187,-0.2494110771,2.1634148225\H,-2.0830179608,-0

.4347318732,1.5824781804\H,-2.216277722,-0.3599518793,4.0522371988\C,-

0.184253625,0.005392368,5.8201617498\C,0.1424391735,1.1301302387,6.654

3112548\C,0.3785633284,2.4675018298,6.1829396134\C,0.7405060003,3.4696

164122,7.0582511789\C,0.8495081255,3.2254075645,8.4393639354\C,0.57237

29099,1.9800688156,8.9427941262\C,0.2063585997,0.9234322956,8.07065282

39\C,-0.1197781168,-0.3509047375,8.6098698784\C,-0.4957749566,-1.38664

77941,7.7986412272\C,-0.5137444952,-1.2026079622,6.3983204519\H,-0.751

4867445,-2.0403482552,5.7488880754\H,-0.7533194189,-2.3537320706,8.218

2970772\H,-0.0708894174,-0.4796162479,9.6876059168\H,0.6206586045,1.78

28919904,10.0093221926\H,1.1325682702,4.0368505852,9.1023176633\H,0.91

30085184,4.4582720058,6.647071741\O,0.2389425931,2.8537402836,4.888210

3769\H,-0.0336545817,2.1226503484,4.3204943349\H,1.9982181722,0.406351

7951,4.2550045061\H,2.1310326728,0.3327570717,1.7887468989\H,0.2594615

988,0.9410696353,-0.3947731873\H,0.9639171541,-0.6764190092,-0.3383511

7\H,-0.7928475837,-0.4789915055,-0.4447050723\\Version=EM64L-G09RevA.0

2\State=1-A\HF=-731.208542\RMSD=5.664e-09\RMSF=2.342e-06\Dipole=-0.087

5063,-0.6772182,-0.5390229\Quadrupole=-3.8347447,-4.4901735,8.3249181,

2.7728985,0.6657843,1.3209613\PG=C01 [X(C17H14O1)]\\@

**4(b)**

1\1\GINC-ABE0579\FOpt\RM062X\6-31+G(d,p)\C17H14O1\ACROFT\29-Jan-2011\0

\\#P M062X/6-31+G(d,p) OPT(EstmFC) FREQ=noraman int=ultrafine\\methyln

aphthol OH out\\0,1\C,-0.0845778857,-0.0767532459,-0.0104004076\C,-0.0

26808682,-0.0481426173,1.496305749\C,1.1867092292,-0.1095510399,2.1797

009618\C,1.2283795991,-0.090013705,3.5749876506\C,0.0544678026,0.00442

0356,4.3211399301\C,-1.1667498754,0.062168963,3.6386265284\C,-1.204464

9563,0.0350567798,2.2514944044\H,-2.1634504247,0.0766165595,1.73980304

29\H,-2.0897950512,0.1328579594,4.207830534\C,0.0951250977,-0.06180528

42,5.811219949\C,-0.3957824631,0.9673688384,6.6839620275\C,-0.82207954

85,2.2644641643,6.2460354838\C,-1.3151156115,3.1914165784,7.1369477856

\C,-1.3860822308,2.9039119385,8.5165816426\C,-0.9426815418,1.697552775

9,8.9878085186\C,-0.4318037004,0.7192853629,8.0918263683\C,0.066720529

,-0.5093138777,8.5998460997\C,0.5872626757,-1.4549598007,7.757356368\C

,0.5883728203,-1.2273328961,6.3641786665\H,0.9514633948,-2.0021148753,

5.6952149003\H,0.9769314495,-2.3890024491,8.1496122606\H,0.0335485689,

-0.6764742268,9.6727914358\H,-0.9660887576,1.4700357098,10.0491257315\

H,-1.7776259663,3.6535911502,9.1965645697\H,-1.6348547561,4.1632609301

,6.7685108327\O,-0.6961329467,2.5613086722,4.9246800694\H,-0.975627406

7,3.4713359636,4.7717243816\H,2.1842633403,-0.1345686959,4.0899673464\

H,2.1149141468,-0.1740360707,1.6174288149\H,0.9177859075,-0.1181330587

,-0.4432703925\H,-0.6419792036,-0.9494427243,-0.3654538878\H,-0.585632

1473,0.8140552147,-0.4020797786\\Version=EM64L-G09RevA.02\State=1-A\HF

=-731.2040786\RMSD=7.232e-09\RMSF=2.047e-06\Dipole=-0.2152848,0.517008

7,-0.0424751\Quadrupole=-4.6318263,1.6329257,2.9989006,-5.0912251,-0.1

415498,-0.8765068\PG=C01 [X(C17H14O1)]\\@

**5(a)**

1\1\GINC-ABE0540\FOpt\RM062X\6-31+G(d,p)\C17H14O2\ACROFT\01-Aug-2010\0

\\#p M062X/6-31+G(d,p) opt freq=noraman int=ultrafine\\1-methoxyphenyl

-8-napthol radical M06-2X/6-31+G(d,p) opt//b3lyp/6-31G(d)\\0,1\C,0.002

3216827,-0.0506359844,-0.0036391373\O,0.0030777025,0.0207938514,1.4081

701132\C,1.2028480431,0.019137463,2.0420417374\C,1.1533982167,0.085676

8675,3.4412309107\C,2.3258689533,0.0892570562,4.1781003594\C,3.5810289

533,0.0212915312,3.5458508295\C,3.6115199921,-0.0400317941,2.15185133\

C,2.4377251288,-0.0450203824,1.3940752655\H,2.5036852529,-0.1012900401

,0.3143183731\H,4.5716873674,-0.0944156839,1.6463138647\C,4.8419164353

,0.1034965874,4.3423936162\C,5.2386744512,-0.8801035488,5.313527938\C,

4.5933583271,-2.148859987,5.5137274447\C,5.0117967179,-3.0031165907,6.

5119709818\C,6.1056425392,-2.6792447873,7.3351953082\C,6.7926801172,-1

.5083789625,7.1394288113\C,6.3838633168,-0.6019353563,6.1282778567\C,7

.1301081264,0.5877963602,5.907658792\C,6.7725765102,1.4748723383,4.929

1048875\C,5.6148999632,1.2307521374,4.1574119175\H,5.2930843985,1.9647

573495,3.4241075507\H,7.3526220044,2.3763660413,4.7599205269\H,7.99944

33405,0.7716317775,6.5329202239\H,7.6585764475,-1.2584350641,7.7447862

257\H,6.4127261926,-3.3748124511,8.109788395\H,4.4839654444,-3.9440514

598,6.6229033019\O,3.5732442824,-2.6112576439,4.7453293334\H,3.3434882

429,-1.9902215114,4.0431034682\H,2.2810252631,0.1414279114,5.262626035

2\H,0.1828233445,0.1371767053,3.9227481055\H,0.5201756642,0.8099243279

,-0.4430097798\H,0.4714171228,-0.9780278662,-0.3523307405\H,-1.0442604

853,-0.0373751636,-0.3045954701\\Version=EM64L-G09RevA.02\State=1-A\HF

=-806.3924657\RMSD=7.027e-09\RMSF=4.751e-06\Dipole=-0.1089257,0.597619

4,-1.0138626\Quadrupole=-0.8952532,-6.7300538,7.625307,1.5150561,7.803

4546,-2.1038632\PG=C01 [X(C17H14O2)]\\@

**5(b)**

1\1\GINC-ABE0539\FOpt\RM062X\6-31+G(d,p)\C17H14O2\ACROFT\01-Aug-2010\0

\\#p M062X/6-31+G(d,p) opt freq=noraman int=ultrafine\\1-methoxyphenyl

-8-napthol radical M06-2X/6-31+G(d,p) opt//b3lyp/6-31G(d)\\0,1\C,-0.02

15122082,-0.0286973686,0.0224868469\O,-0.0043709131,-0.009646927,1.433

598835\C,1.2105799384,-0.0014299623,2.0494642475\C,1.1831324033,0.0274

04542,3.4504567401\C,2.3663833186,0.0323280289,4.1667961932\C,3.608320

2255,0.0122662039,3.5139991808\C,3.6192658046,-0.0032892294,2.12299756

43\C,2.4333413317,-0.0165138699,1.381129526\H,2.4842587232,-0.03918424

67,0.2991930279\H,4.5712901794,-0.0206657838,1.599415697\C,4.881962771

7,0.1089197325,4.2836910061\C,5.314956398,-0.8512534295,5.2606146212\C

,4.6727321324,-2.1111806074,5.4987507588\C,5.1228887532,-2.9673422474,

6.4789006604\C,6.2529620644,-2.6451421134,7.2597481433\C,6.9299293293,

-1.4770584049,7.0334657206\C,6.4870862971,-0.5716660405,6.031029158\C,

7.2269457651,0.6133468873,5.7771541805\C,6.8332147703,1.4876988844,4.7

997461263\C,5.6541667374,1.2334937759,4.066444786\H,5.3139745422,1.957

9881277,3.3323022789\H,7.4058922812,2.3882201999,4.6015077537\H,8.1165

928568,0.8044110608,6.3706796414\H,7.8166653438,-1.2255781134,7.607194

8062\H,6.5864295648,-3.3389976503,8.0246458027\H,4.6071248547,-3.91177

36877,6.635518503\O,3.6205151045,-2.4489678019,4.7055282025\H,3.306629

088,-3.3284526858,4.9457544343\H,2.3379050503,0.0490266007,5.252800374

1\H,0.2190764037,0.0480603371,3.9478369283\H,0.4715157453,0.8588854309

,-0.3920792866\H,0.465332436,-0.9304207985,-0.3682139882\H,-1.07173083

81,-0.0290001983,-0.2674140852\\Version=EM64L-G09RevA.02\State=1-A\HF=

-806.3880237\RMSD=7.256e-09\RMSF=3.135e-06\Dipole=0.0533343,-0.5039548

,-0.251087\Quadrupole=-3.8893104,-0.4022985,4.2916089,3.9579313,5.6207

568,-4.8967626\PG=C01 [X(C17H14O2)]\\@

**6**

1\1\GINC-ABE0243\FOpt\RM062X\6-31+G(d,p)\C10H8O1\ACROFT\02-Feb-2011\0\

\#P M062X/6-31+G(d,p) OPT(EstmFC) FREQ=noraman int=ultrafine\\napthol

in\\0,1\C,0.0602602371,-0.07651231,-0.028919429\H,0.1939432236,-0.2188

737266,1.0377477734\C,1.2087387162,-0.0341906066,-0.8607171085\C,2.524

9986319,-0.1736971386,-0.3290308195\C,3.626169929,-0.1303320341,-1.149

4304912\C,3.4632353643,0.055148335,-2.5440241052\C,2.2150667049,0.1932

912548,-3.0935164605\C,1.061175625,0.1514236434,-2.2628351721\C,-0.250

5967896,0.2902917382,-2.7905661132\C,-1.3496885213,0.2465970717,-1.969

0135578\C,-1.1931917759,0.0612787976,-0.5735902028\H,-2.0696930251,0.0

283651832,0.0655679206\H,-2.345388881,0.3544428905,-2.3878024681\H,-0.

3673929997,0.4322307889,-3.861572388\H,2.09063766,0.3355595218,-4.1627

423428\H,4.3451817205,0.0866176748,-3.1757871004\H,4.622691897,-0.2383

003341,-0.7286335488\O,2.607944778,-0.3492841786,1.0199096952\H,3.5312

181503,-0.4331346295,1.2831972498\\Version=EM64L-G09RevA.02\State=1-A\

HF=-460.9425831\RMSD=8.647e-09\RMSF=1.922e-05\Dipole=0.503435,-0.02784

12,0.0025905\Quadrupole=6.3555704,-7.4849623,1.1293919,-1.1318501,2.84

2856,-1.2743648\PG=C01 [X(C10H8O1)]\\@

**6 anion**

1\1\GINC-ABE0538\FOpt\RM062X\6-31+G(d,p)\C10H7O1(1-)\ACROFT\29-Jan-201

1\0\\#P M062X/6-31+G(d,p) OPT(EstmFC) FREQ=noraman int=ultrafine\\napt

hololate\\-1,1\C,0.0368759844,0.,-0.0382902156\H,0.1970973083,0.,1.036

8145321\C,1.1919412107,0.,-0.8455573865\C,2.5178580112,0.,-0.192585314

9\C,3.6259415368,0.,-1.0952106315\C,3.4564704974,0.,-2.4817076975\C,2.

2083009572,0.,-3.0911404314\C,1.0539564822,0.,-2.267562631\C,-0.265853

8662,0.,-2.8056988003\C,-1.3768436384,0.,-1.992914479\C,-1.2313062641,

0.,-0.5855815894\H,-2.1106840612,0.,0.0534578085\H,-2.3713210461,0.,-2

.4347925144\H,-0.3805386822,0.,-3.8887733872\H,2.1031144446,0.,-4.1728

969835\H,4.3465966526,0.,-3.1119308883\H,4.6209387476,0.,-0.6579438049

\O,2.6269372528,0.,1.0628325062\\Version=EM64L-G09RevA.02\State=1-A'\H

F=-460.388352\RMSD=9.628e-09\RMSF=9.171e-05\Dipole=-1.347752,0.,-1.606

1269\Quadrupole=-0.3402004,4.303048,-3.9628476,0.,-5.6722201,0.\PG=CS

[SG(C10H7O1)]\\@

**8**

1\1\GINC-ABE0547\FOpt\RM062X\6-31+G(d,p)\C16H11O1(1-)\ACROFT\28-Apr-20

10\0\\#p M062X/6-31+G(d,p) opt freq=noraman\\1-phenyl-8-napthol anion

M06-2X/6-31+G(d,p) opt//b3lyp/6-31G(d)\\-1,1\C,0.0478011788,-0.5197204

789,0.084344926\C,0.1556287952,-0.5208548026,1.4770718006\C,1.29488321

54,-0.0077402537,2.0878649639\C,2.3206793078,0.5151196703,1.2934635953

\C,2.2034675837,0.526670714,-0.0897428767\C,1.0638658481,0.0055875525,

-0.717851672\C,0.9901113467,-0.1222013169,-2.2047046396\C,1.1677922113

,0.9656880779,-3.1114038672\C,1.2427244641,2.3688425752,-2.6371341502\

C,1.4979229493,3.343918748,-3.6518923636\C,1.5805432676,3.0202400035,-

5.0032711725\C,1.4339130404,1.7184772402,-5.4596380945\C,1.2210261219,

0.6823693865,-4.5178524266\C,1.0424508726,-0.6602668528,-4.9585210176\

C,0.8257620743,-1.6815123142,-4.0686658903\C,0.8086838529,-1.412196356

5,-2.6843507581\H,0.6955934648,-2.2273425889,-1.9744079564\H,0.6878729

043,-2.700700153,-4.4226812229\H,1.0776199817,-0.8583983568,-6.0284303

313\H,1.473332143,1.4783492816,-6.5186165165\H,1.751464389,3.819109644

5,-5.7252255405\H,1.5845319001,4.3742628322,-3.3180860535\O,1.08153092

29,2.6751177594,-1.4274611775\H,2.9851840764,0.9600484697,-0.705221846

\H,3.2103424645,0.9291509303,1.7605799027\H,1.3848130426,-0.0055871917

,3.1709707241\H,-0.6550512612,-0.9213559239,2.0805700825\H,-0.84608309

77,-0.9134200178,-0.3925660212\\Version=EM64L-G09RevA.02\State=1-A\HF=

-691.3534322\RMSD=3.780e-09\RMSF=1.700e-05\Dipole=0.0740013,-1.8371951

,0.5272679\Quadrupole=7.6351337,-3.5535432,-4.0815905,2.2023122,0.8023

541,1.6319077\PG=C01 [X(C16H11O1)]\\@

**9**

1\1\GINC-ABE0541\FOpt\RM062X\6-31+G(d,p)\C16H10N1O3(1-)\ACROFT\01-Aug-

2010\0\\#p M062X/6-31+G(d,p) opt freq=noraman int=ultrafine guess=chec

k geom=check\\1-nitrophenyl-8-napthol anion M06-2X/6-31+G(d,p) opt//b3

lyp/6-31G(d)\\-1,1\C,0.2255058857,0.5020109262,-0.0681661987\C,0.11931

11505,0.4818739336,1.3100787556\C,1.1827859704,-0.0297567495,2.0582567

429\C,2.3359947912,-0.5170179489,1.4569058527\C,2.4157061457,-0.501057

3993,0.0683948933\C,1.3720009512,0.0075923498,-0.7143717716\C,1.420270

3638,-0.1171891996,-2.1980526841\C,1.1872402736,0.9712949425,-3.088346

2514\C,1.1051828633,2.3607859326,-2.5901613926\C,0.8009815818,3.345101

6703,-3.579050883\C,0.6758531308,3.0350683854,-4.9313647532\C,0.828540

6681,1.7421699299,-5.4116358825\C,1.0925598078,0.6990209997,-4.4928992

361\C,1.285789352,-0.6372623665,-4.9474708826\C,1.5580717163,-1.659052

9709,-4.0735649699\C,1.6199729586,-1.3999305941,-2.6876780895\H,1.7760

738629,-2.2178617631,-1.9892627112\H,1.7070734433,-2.6717254808,-4.439

6434292\H,1.2197988852,-0.8300564289,-6.0165791011\H,0.7590533114,1.51

70283311,-6.4720412408\H,0.4663347165,3.8398054071,-5.6356640541\H,0.7

080784088,4.3696461453,-3.2305026342\O,1.3024071803,2.6408311492,-1.37

74674639\H,3.3100396037,-0.8704129646,-0.4236843247\H,3.1431799492,-0.

8978135969,2.0707314937\N,1.0783058661,-0.0607441009,3.5130621435\O,2.

0239366218,-0.5021317354,4.1540997073\O,0.0480389164,0.3509761133,4.02

88258771\H,-0.7588262443,0.8608150545,1.8179707173\H,-0.5731416307,0.9

193850589,-0.6709325875\\Version=EM64L-G09RevA.02\State=1-A\HF=-895.80

32533\RMSD=9.016e-09\RMSF=1.920e-05\Dipole=0.0511856,-1.8926504,-0.408

4103\Quadrupole=18.5485045,9.3025939,-27.8510984,-1.6895936,0.4637395,

8.1025019\PG=C01 [X(C16H10N1O3)]\\@

**10**

1\1\GINC-ABE0535\FOpt\RM062X\6-31+G(d,p)\C16H10Cl1O1(1-)\ACROFT\29-Jan

-2011\0\\#P M062X/6-31+G(d,p) OPT(EstmFC) FREQ=noraman int=ultrafine\\

chloronaphtholate\\-1,1\C,0.000225076,-0.1906232946,0.0400393836\C,0.0

583401994,-0.2835423772,1.433079655\C,1.2787880232,-0.0987944525,2.061

477884\C,2.4324754143,0.1861752585,1.332878659\C,2.3505492918,0.287324

1201,-0.0485479828\C,1.1349770043,0.0958706666,-0.7205455113\C,1.07237

56963,0.0594571041,-2.211717719\C,1.5644314715,1.0995229527,-3.0565543

275\C,2.0022976404,2.4048546006,-2.5086693107\C,2.5404490577,3.3198310

844,-3.4661492279\C,2.5722583087,3.0485254119,-4.8313802761\C,2.092381

6892,1.8578875414,-5.3573409947\C,1.580227711,0.8766237231,-4.47479307

57\C,1.0581822099,-0.3456805717,-4.987448113\C,0.5483086651,-1.3099532

707,-4.1561377316\C,0.5641881321,-1.1100419033,-2.7598073678\H,0.21726

23897,-1.8976754088,-2.0961111381\H,0.15158184,-2.236369161,-4.5645131

287\H,1.0691196473,-0.4957088623,-6.0654873717\H,2.0956204438,1.665326

945,-6.4265743187\H,2.9741554615,3.8032181764,-5.5074451595\H,2.893101

3576,4.2710285199,-3.0774571947\O,1.8965986452,2.6860335699,-1.2858031

012\H,3.2339271359,0.5381482777,-0.6256790033\H,3.3748694838,0.3396777

066,1.8476938897\Cl,1.3770172008,-0.2262119891,3.8141833305\H,-0.83120

85471,-0.4942799154,2.0174723964\H,-0.9527417383,-0.3250566392,-0.4638

704762\\Version=EM64L-G09RevA.02\State=1-A\HF=-1150.9282669\RMSD=4.135

e-09\RMSF=7.615e-06\Dipole=-0.4883395,-1.922339,0.7012283\Quadrupole=1

3.8798779,0.5467399,-14.4266177,-0.8133442,1.9437585,5.9190036\PG=C01

[X(C16H10Cl1O1)]\\@

**11**

1\1\GINC-ABE0536\FOpt\RM062X\6-31+G(d,p)\C17H13O1(1-)\ACROFT\29-Jan-20

11\0\\#P M062X/6-31+G(d,p) OPT(EstmFC) FREQ=noraman int=ultrafine\\met

hylnaphtholate\\-1,1\C,0.0306070093,0.0376122508,0.0055070387\C,0.0144

10225,0.0051415696,1.5143150769\C,1.2146496319,-0.0451258408,2.2391249

975\C,1.2189636281,-0.0488182013,3.6251195004\C,0.0176574189,-0.017077

3576,4.3490690689\C,-1.175904063,0.0229142613,3.6294815382\C,-1.177866

9031,0.0413882857,2.2309908513\H,-2.1244814102,0.0880749212,1.69554382

43\H,-2.1171694617,0.0601573579,4.1717570083\C,0.0044663697,-0.1645795

178,5.8352044851\C,0.7471185733,0.6654903514,6.7289921116\C,1.44508919

02,1.8876092219,6.260980326\C,2.2143099456,2.5709805529,7.2548595973\C

,2.2369250177,2.1799438097,8.5906816067\C,1.5205716195,1.0839758181,9.

0490946892\C,0.7667634401,0.3221518455,8.1232864719\C,0.0030397589,-0.

793873121,8.5710177583\C,-0.7412283713,-1.5462592478,7.6985675783\C,-0

.731822104,-1.2360045661,6.3227190318\H,-1.2706435645,-1.8673534306,5.

6211382825\H,-1.3207356651,-2.3937802551,8.0574315511\H,0.0208989915,-

1.0347858013,9.6324690506\H,1.52084506,0.8014913994,10.0982849947\H,2.

8249218003,2.7637109087,9.2992572115\H,2.7568445934,3.4512505625,6.921

2719823\O,1.356349388,2.2978486291,5.0746186144\H,2.1568371689,-0.0555

526422,4.1709158081\H,2.1606604635,-0.0651865177,1.7007816753\H,0.4971

173782,0.9573288127,-0.3650558081\H,0.597921687,-0.8049153726,-0.40540

27003\H,-0.9843537178,-0.0096941042,-0.400216566\\Version=EM64L-G09Rev

A.02\State=1-A\HF=-730.6515219\RMSD=6.372e-09\RMSF=1.727e-06\Dipole=-0

.8875252,-1.6662353,-1.3139158\Quadrupole=7.0464752,-2.0387269,-5.0077

483,-3.535511,-3.3115531,-2.0901452\PG=C01 [X(C17H13O1)]\\@

**12**

1\1\GINC-ABE0546\FOpt\RM062X\6-31+G(d,p)\C17H13O2(1-)\ACROFT\01-Aug-20

10\0\\#p M062X/6-31+G(d,p) opt freq=noraman int=ultrafine\\1-methoxyph

enyl-8-napthol anion M06-2X/6-31+G(d,p) opt//b3lyp/6-31G(d)\\-1,1\C,-0

.0205779864,-0.0550860124,0.023609799\O,-0.0014457194,-0.0171187349,1.

427277848\C,1.2333003617,0.0023877677,2.0339895085\C,1.2254718935,-0.0

108648264,3.4347006814\C,2.4168105879,-0.0034278658,4.1362270125\C,3.6

541886178,0.0361584485,3.4706256275\C,3.6395476499,0.0575801416,2.0806

389556\C,2.4423651758,0.0326477486,1.3496393786\H,2.4817453435,0.03304

21149,0.2661896295\H,4.584538574,0.0725590518,1.5441226885\C,4.9367448

187,0.1847764762,4.2209978139\C,5.3522059092,-0.6841015899,5.275988048

5\C,4.6220016122,-1.9362000819,5.5909348967\C,5.111988773,-2.662717241

3,6.7215504202\C,6.2476194049,-2.2795413641,7.4293470614\C,6.979401746

,-1.1507761922,7.0907723841\C,6.5407638267,-0.348467339,6.009321474\C,

7.2880983866,0.8021973573,5.6262330664\C,6.8922749289,1.5947001282,4.5

792292395\C,5.7051563549,1.2902594344,3.8811883383\H,5.3561855322,1.95

10716913,3.0920473585\H,7.47527622,2.4689145462,4.2985047576\H,8.19134

41524,1.0369496167,6.1866364641\H,7.880270806,-0.8726376077,7.63083686

68\H,6.5778951674,-2.895054581,8.2663923033\H,4.5695460999,-3.56712610

02,6.9828170934\O,3.6454875542,-2.3352106547,4.9042211029\H,2.40804925

04,-0.0482874581,5.220063953\H,0.2690433385,-0.0478228462,3.946675443\

H,0.4480800327,0.8388598438,-0.4098213902\H,0.4935884124,-0.9460817906

,-0.3602830684\H,-1.0711551139,-0.089087869,-0.2692584393\\Version=EM6

4L-G09RevA.02\State=1-A\HF=-805.8351848\RMSD=6.815e-09\RMSF=4.334e-06\

Dipole=-0.9139334,1.7281252,-2.2294011\Quadrupole=-3.4559893,-2.084902

2,5.5408915,1.43899,-6.43574,4.6056777\PG=C01 [X(C17H13O2)]\\@
