# Supplementary material for: Anion–π interactions influence pKa values
Source: Beilstein J Org Chem. 2011 Mar 17;7:320–8. doi: 10.3762/bjoc.7.42 (PMC3079108; doi:10.3762/bjoc.7.42)

Supporting Information

for

Anion–π interactions influence pK_a values

Christopher J. Cadman and Anna K. Croft*

Address: School of Chemistry, University of Wales Bangor, Bangor, Gwynedd, LL57 2UW, United Kingdom. Fax: +44 1248 370 528. Tel: +44 1248 382 375.

Email: Anna K. Croft - a.k.croft@bangor.ac.uk

*Corresponding author

Original spectral data for compounds 1–5

Compound 1: 8-phenyl-1-naphthol

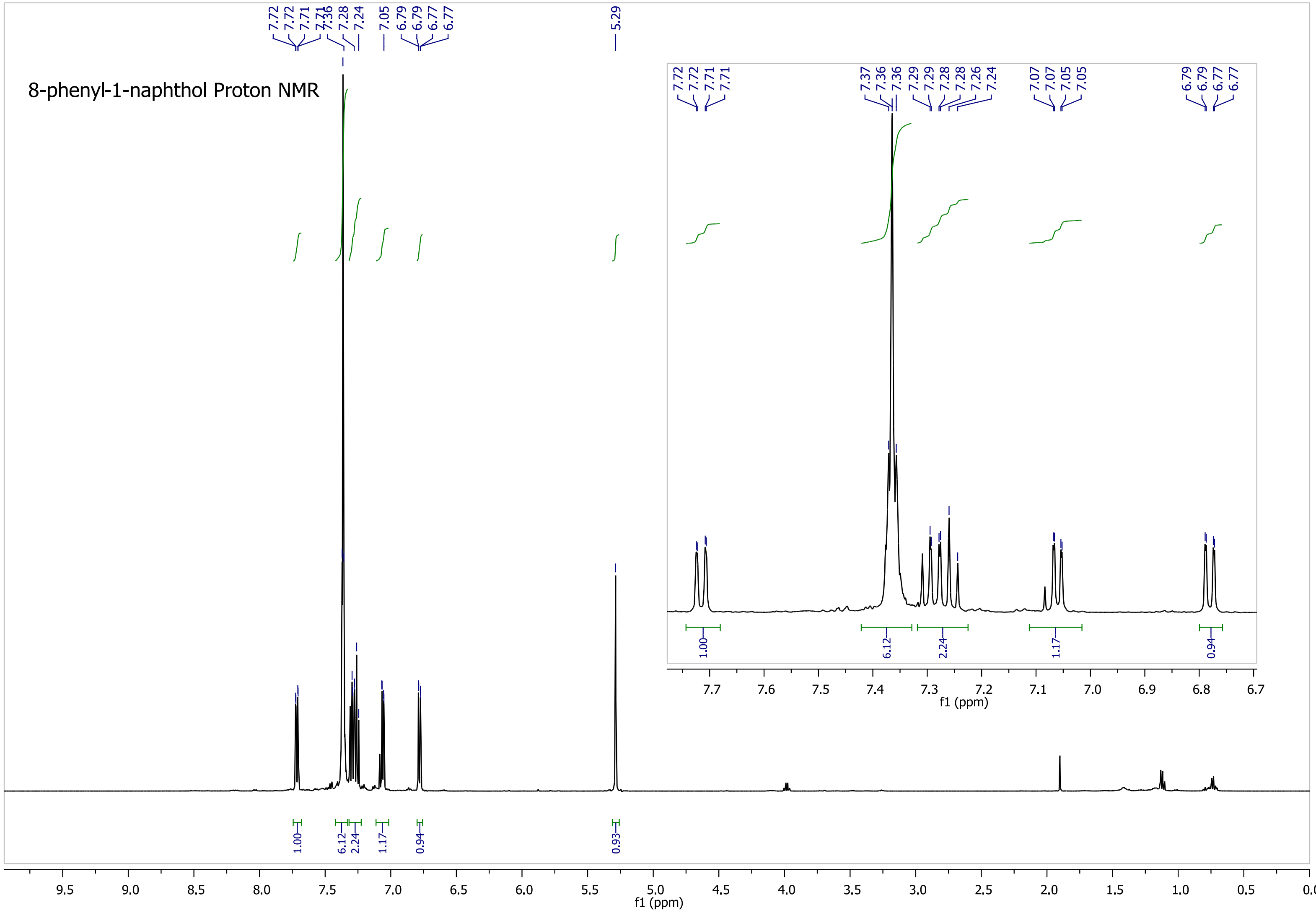

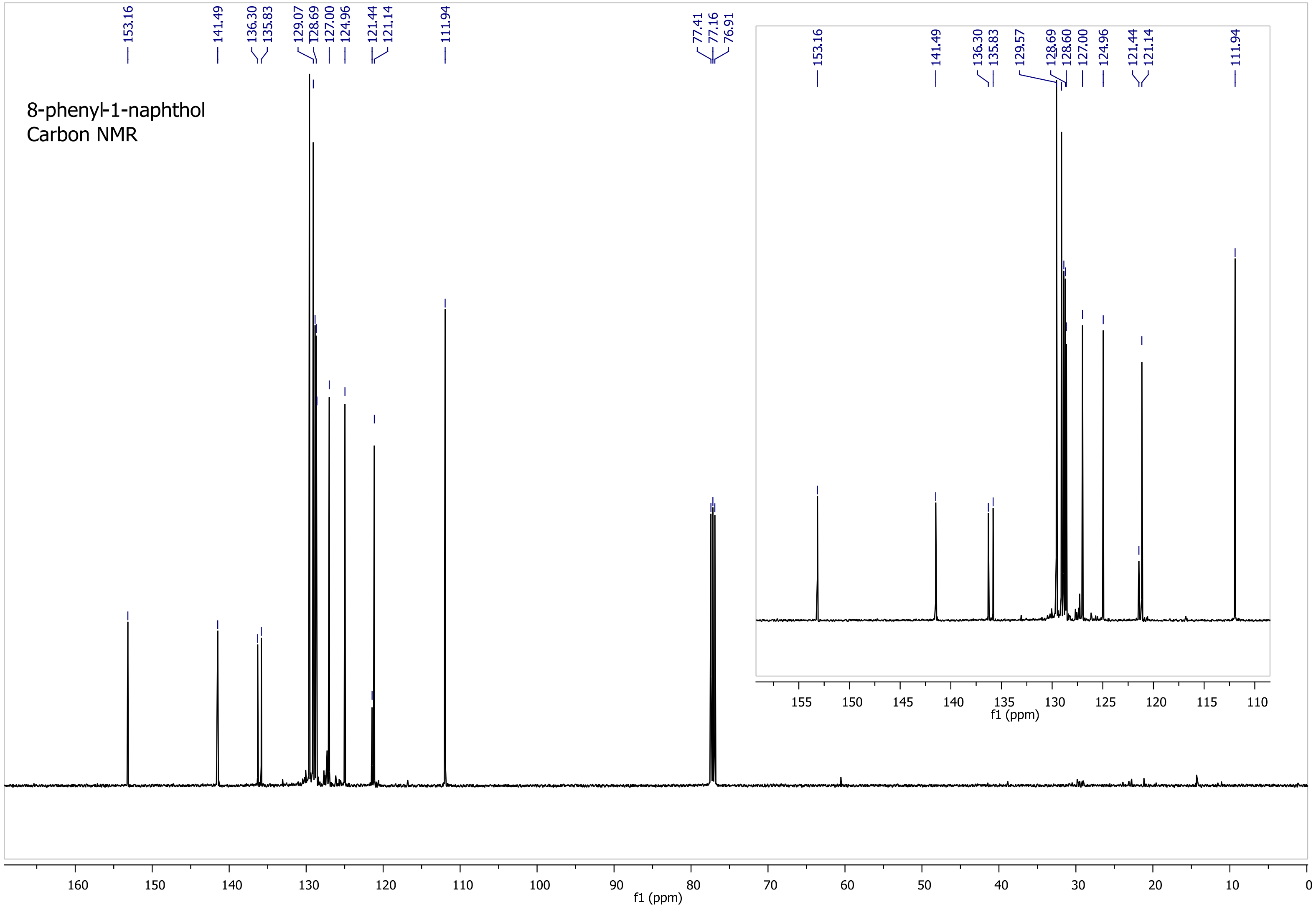

8-phenyl-1-naphthol
Carbon NMR
153.16
141.49
136.30
135.83
129.07
128.69
127.00
124.96
121.44
121.14
111.94
77.41
77.16
76.91
129.57
128.60
f1 (ppm)

Compound 2: 8-(4-nitrophenyl)-1-naphthol

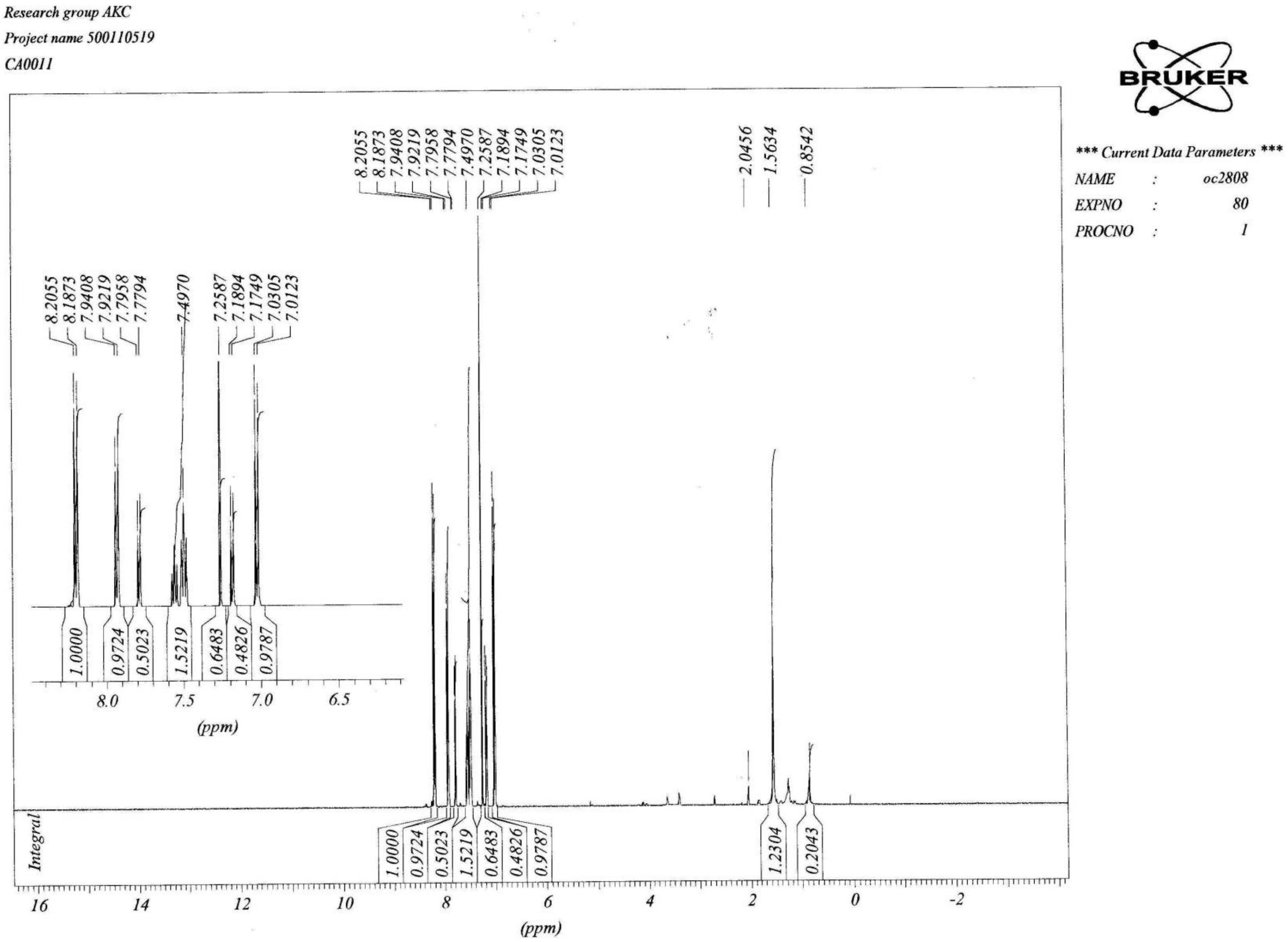

8-(4-nitrophenyl)-1-naphthol Proton NMR

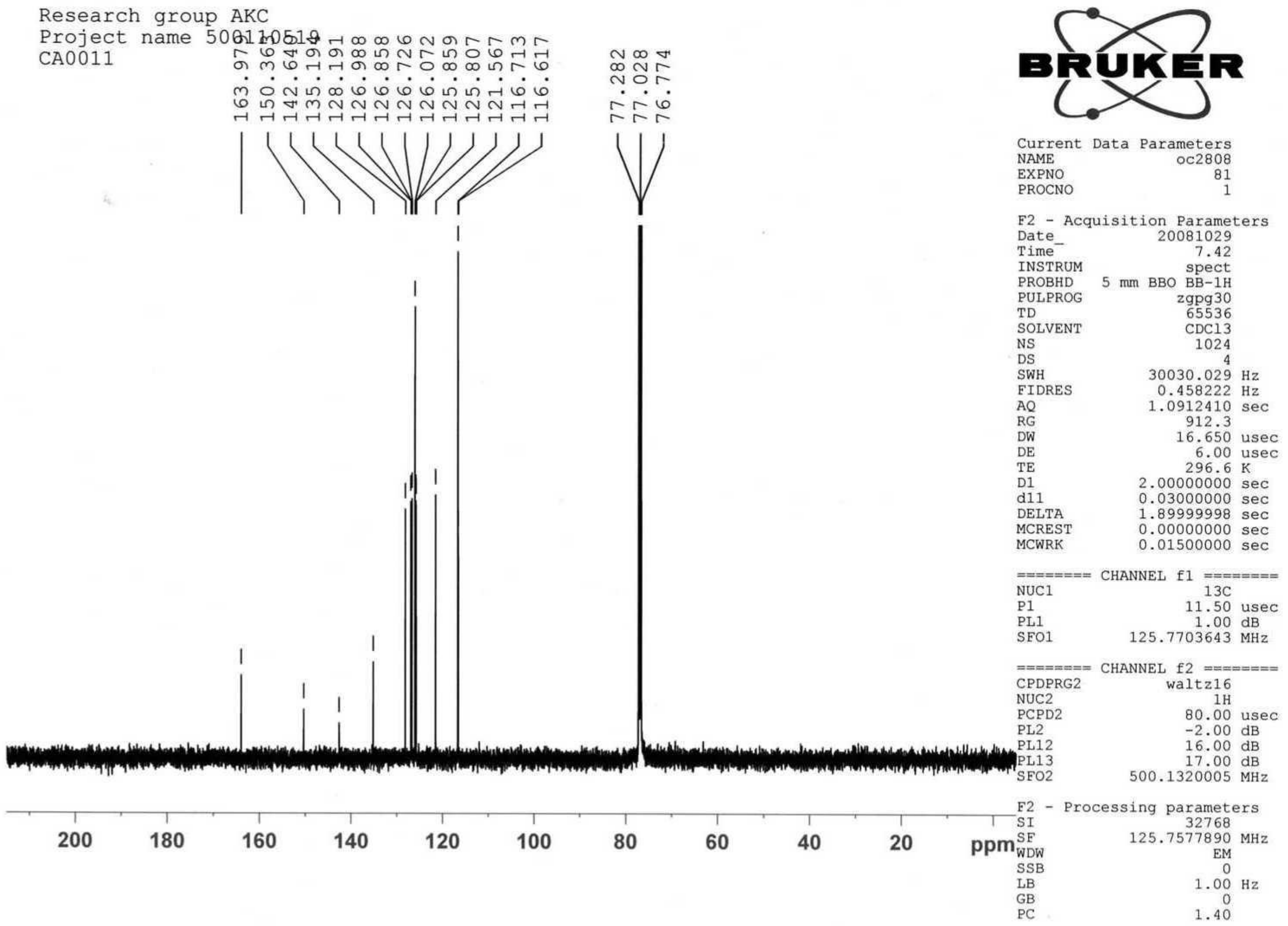

8-(4-nitrophenyl)-1-naphthol Carbon NMR

PerkinElmer Spectrum Express Version 1.02.0
03/11/2008 12:4

Analyst Administrator
Date 03 November 2008 12:43

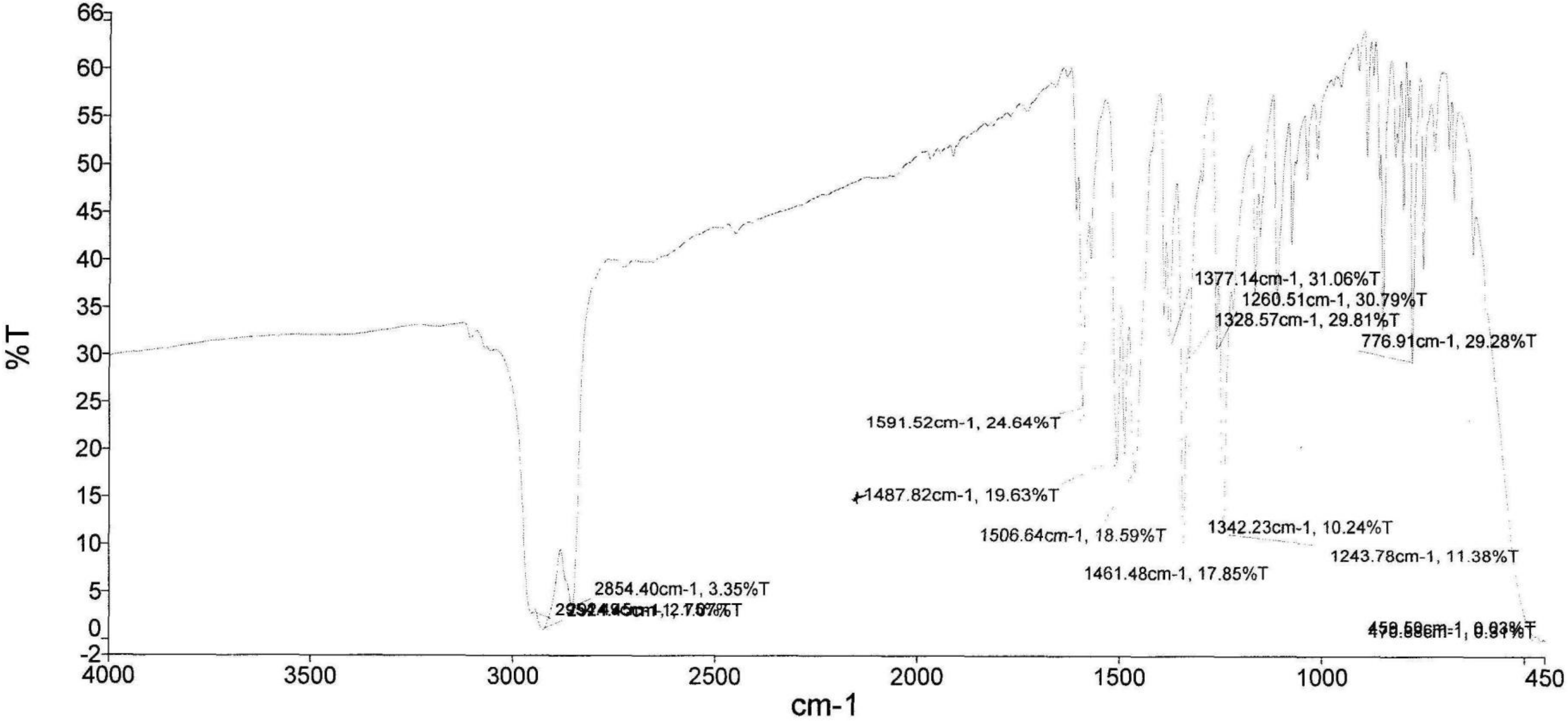

Administrator 62 Sample 062 By Administrator date Monday, November 03 2008

Page

C:\Xcalibur\data\denis\cad02 06/02/2009 13:34:35

cad02 #1299 RT: 24.65 AV: 1 NL: 2.58E5
T: {0,0} + c EI det=350.00 Full ms [50.00-650.00]

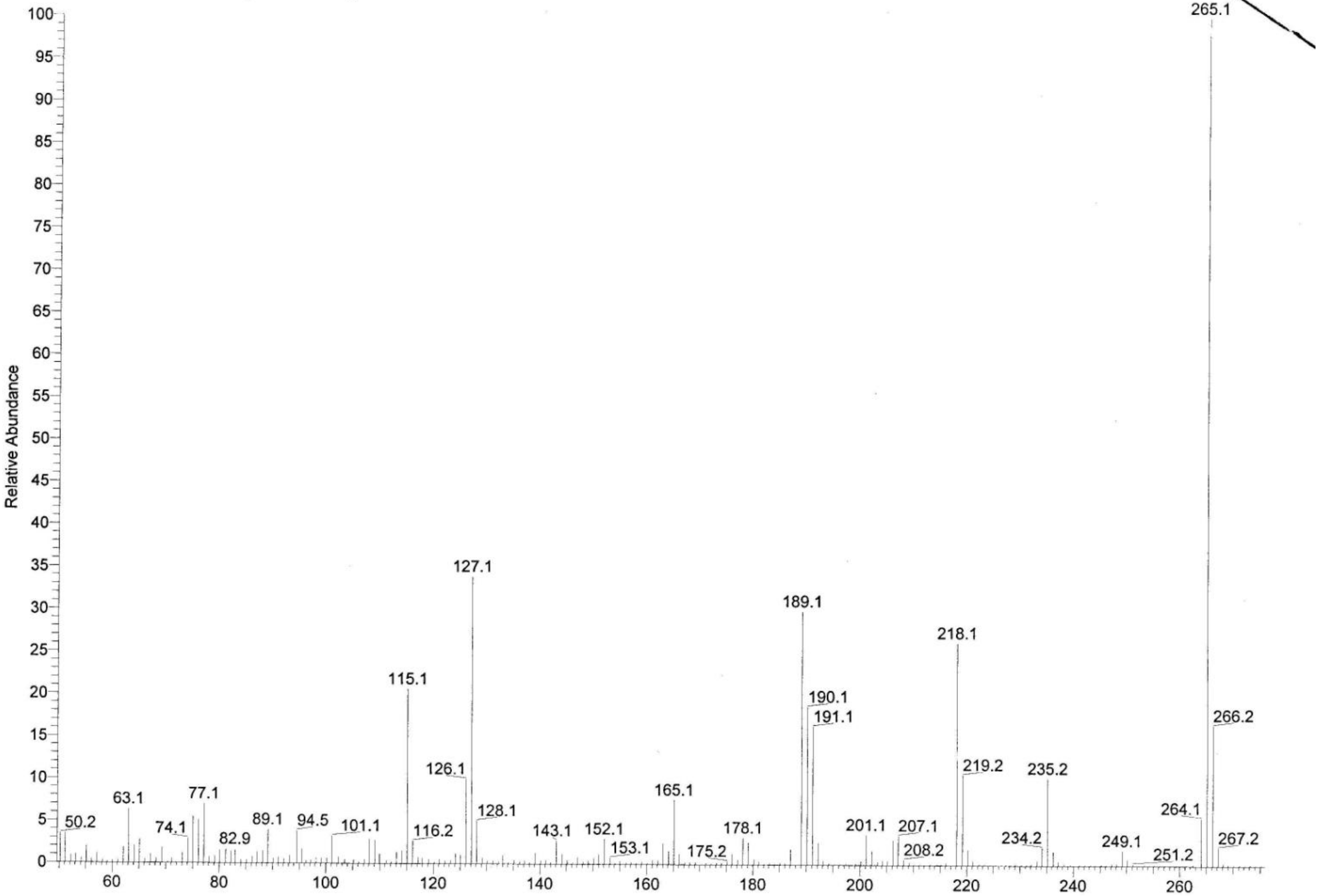

Compound 3: 8-(4-chlorophenyl)-1-naphthol

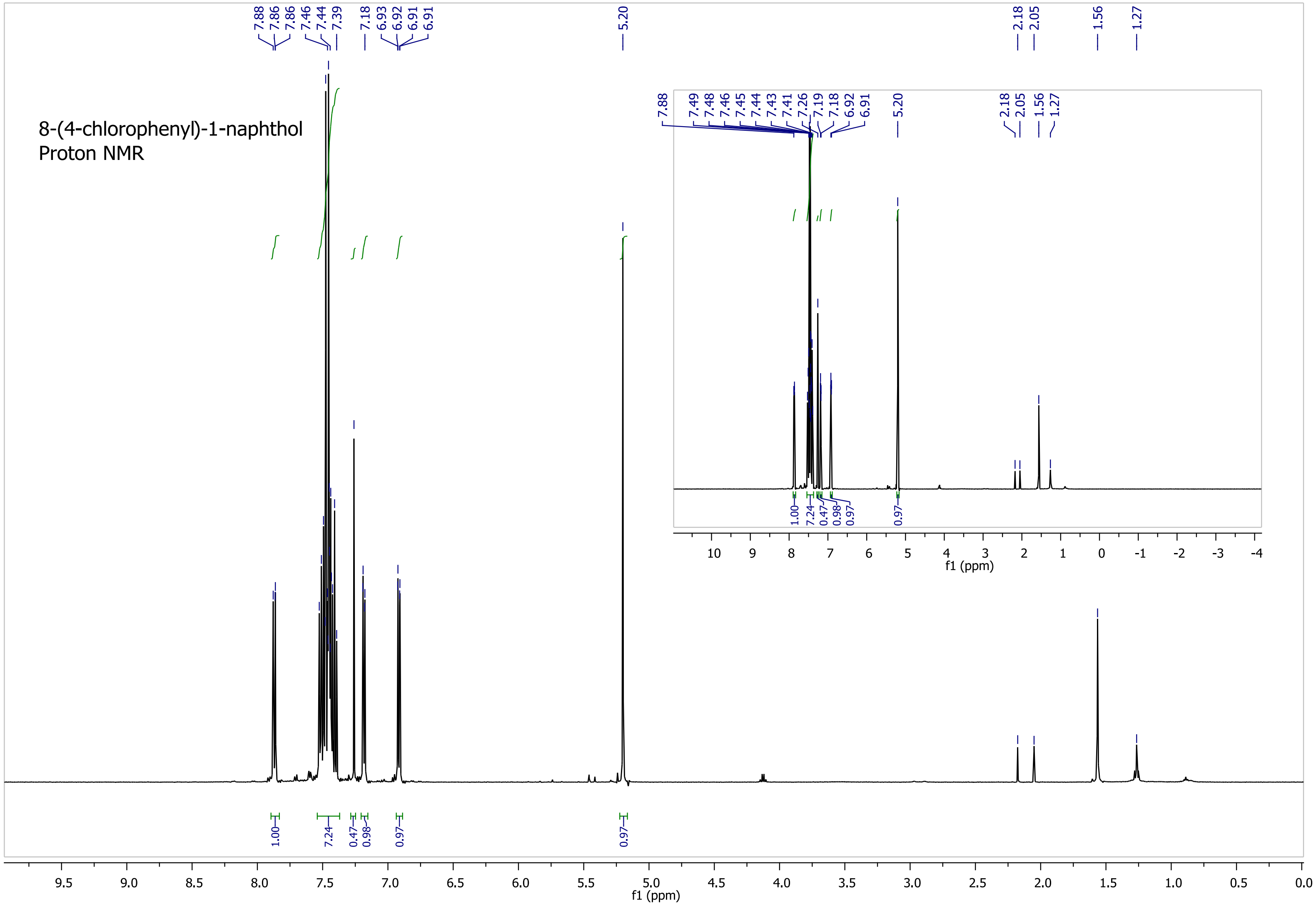

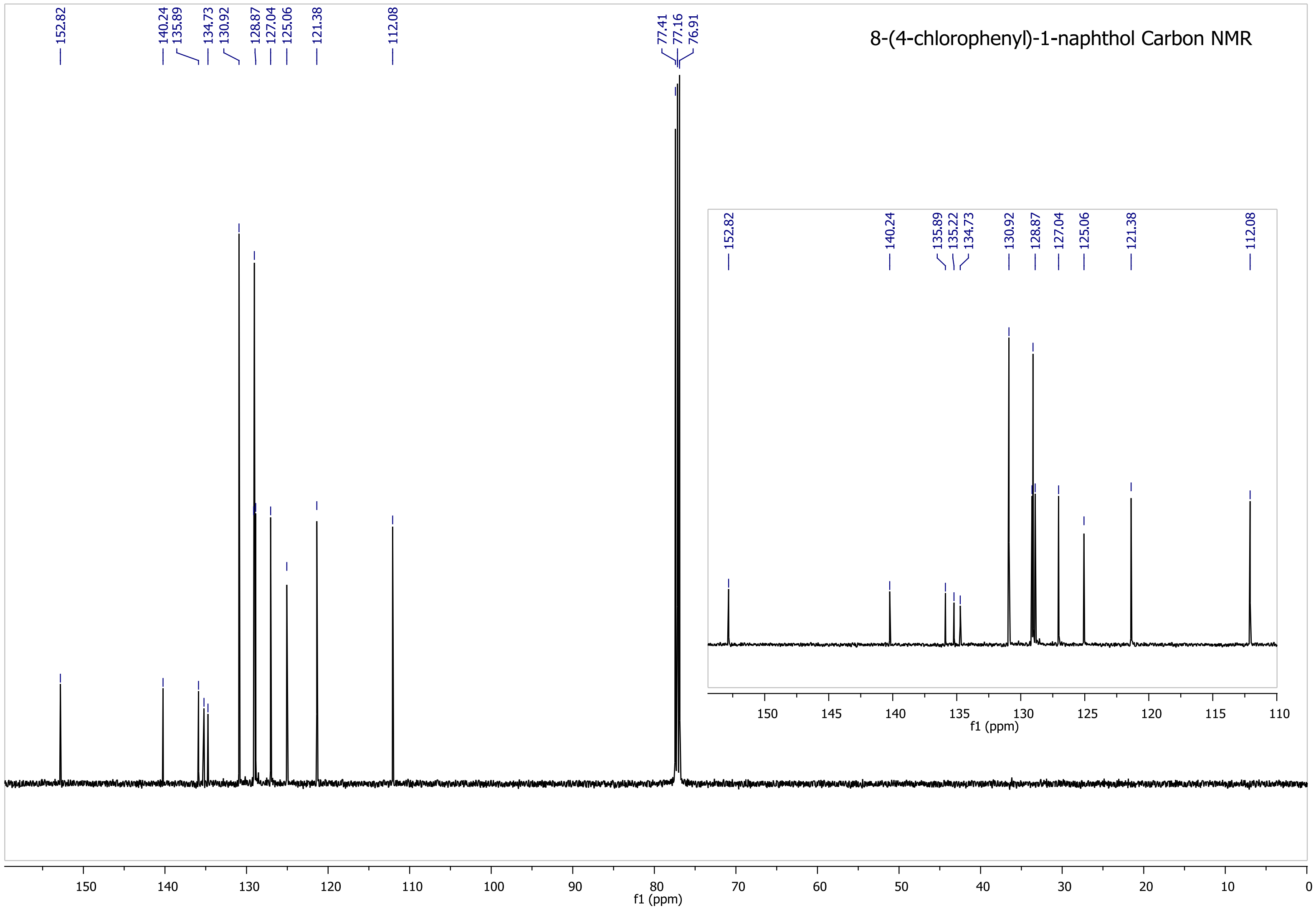
8-(4-chlorophenyl)-1-naphthol Carbon NMR
152.82
140.24
135.89
134.73
130.92
128.87
127.04
125.06
121.38
112.08
77.41
77.16
76.91
152.82
140.24
135.89
135.22
134.73
130.92
128.87
127.04
125.06
121.38
112.08
f1 (ppm)
f1 (ppm)

PerkinElmer Spectrum Express Version 1.02.00
26/01/2009 14:42

Analyst Administrator
Date 26 January 2009 14:42

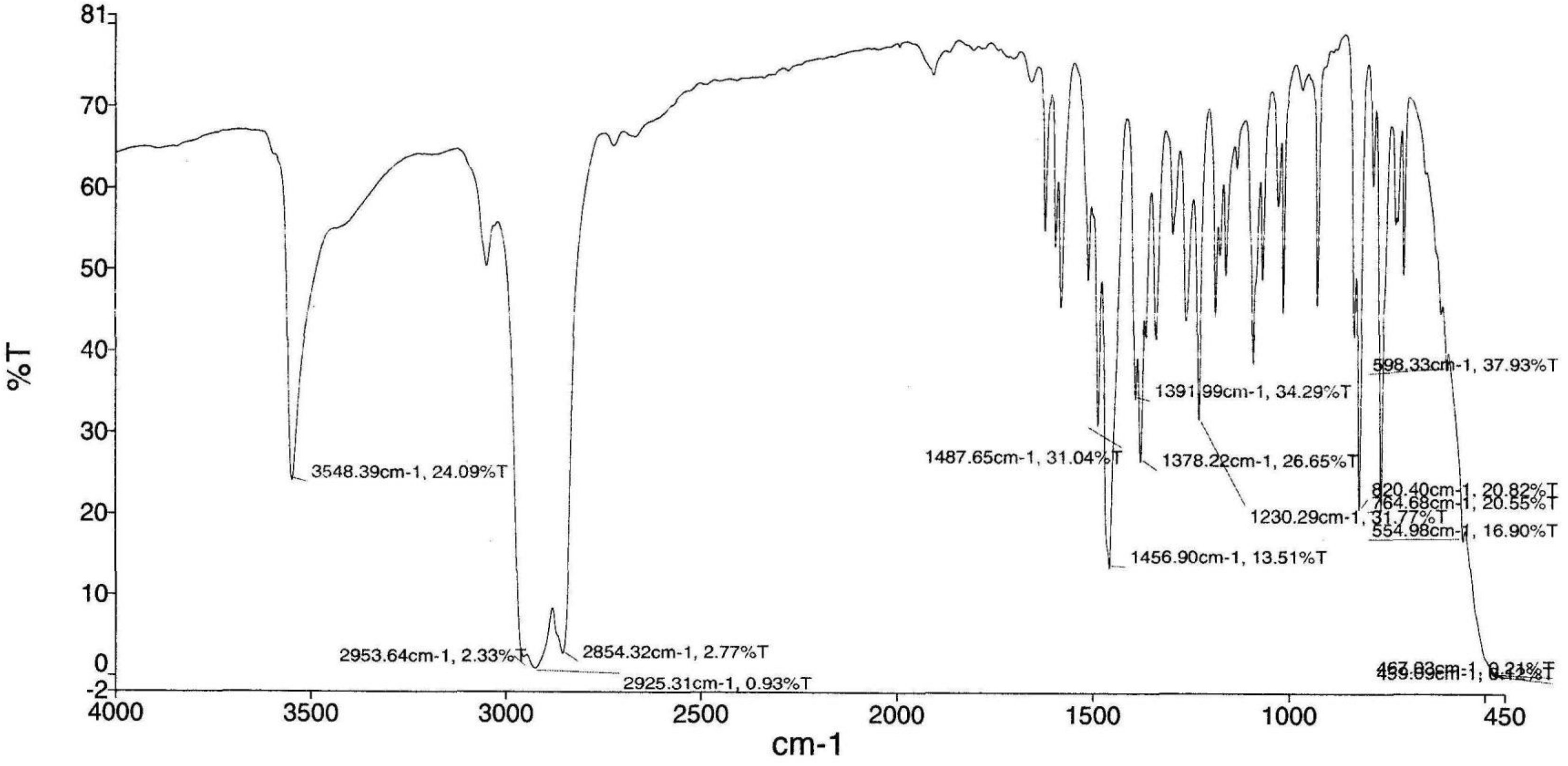

Administrator 10 Sample 010 By Administrator date Monday, January 26 2009

Page 1

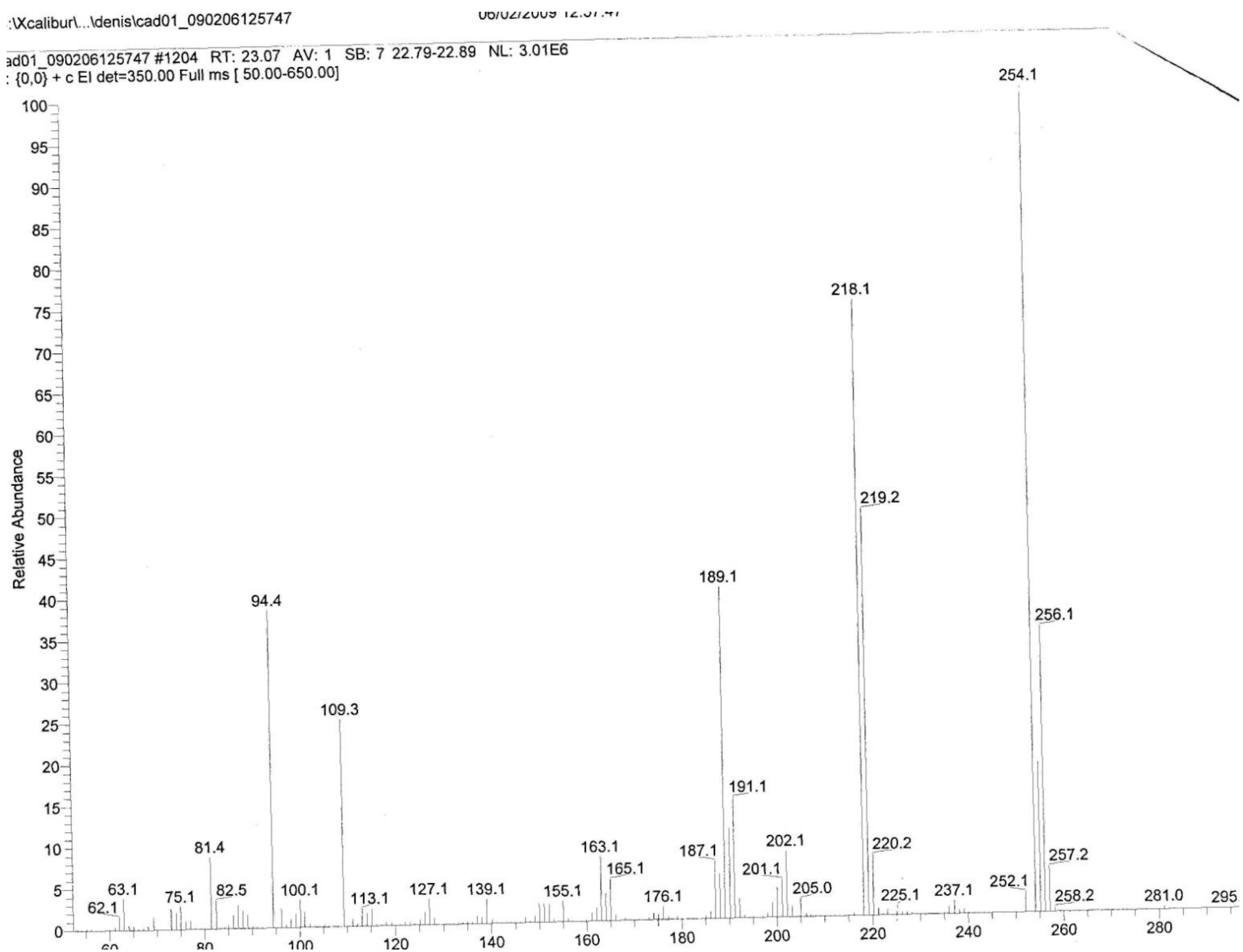
:\Xcalibur\...\denis\cad01_090206125747
06/02/2009 12:57:47
ad01_090206125747 #1204 RT: 23.07 AV: 1 SB: 7 22.79-22.89 NL: 3.01E6
: {0,0} + c EI det=350.00 Full ms [50.00-650.00]
Relative Abundance
254.1
218.1
219.2
189.1
94.4
256.1
109.3
191.1
81.4
163.1
187.1
202.1
220.2
257.2
165.1
201.1
252.1
63.1
75.1
82.5
100.1
127.1
139.1
155.1
205.0
225.1
237.1
62.1
113.1
176.1
258.2
281.0
295

Compound 4: 8-(4-methylphenyl)-1-naphthol

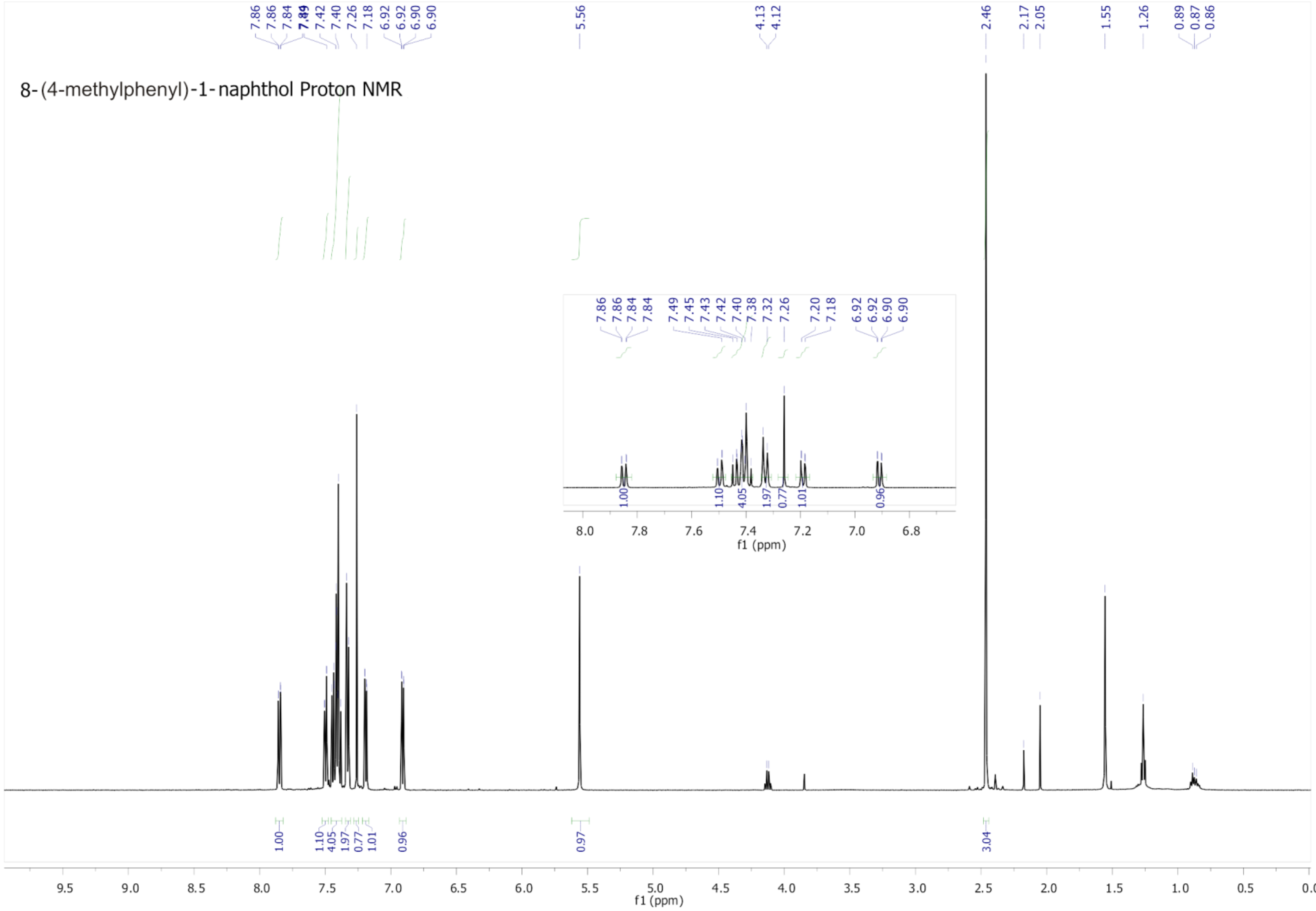

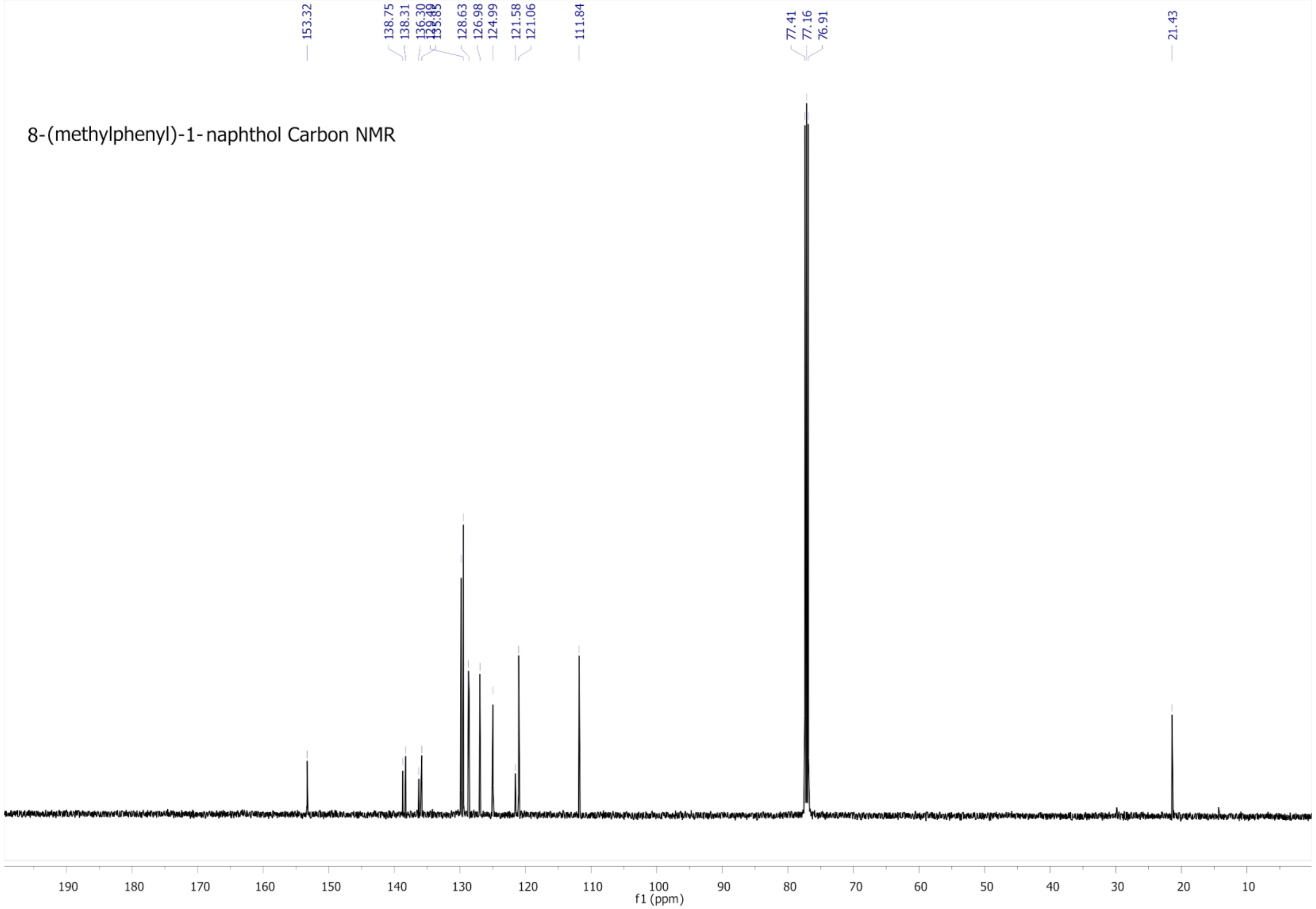
153.32
138.75
138.31
136.30
135.85
128.63
126.98
124.99
121.58
121.06
111.84
77.41
77.16
76.91
21.43
8-(methylphenyl)-1-naphthol Carbon NMR
190
180
170
160
150
140
130
120
110
100
90
80
70
60
50
40
30
20
10
f1 (ppm)

PerkinElmer Spectrum Express Version 1.02.00
26/01/2009 14:54

Analyst Administrator
Date 26 January 2009 14:54

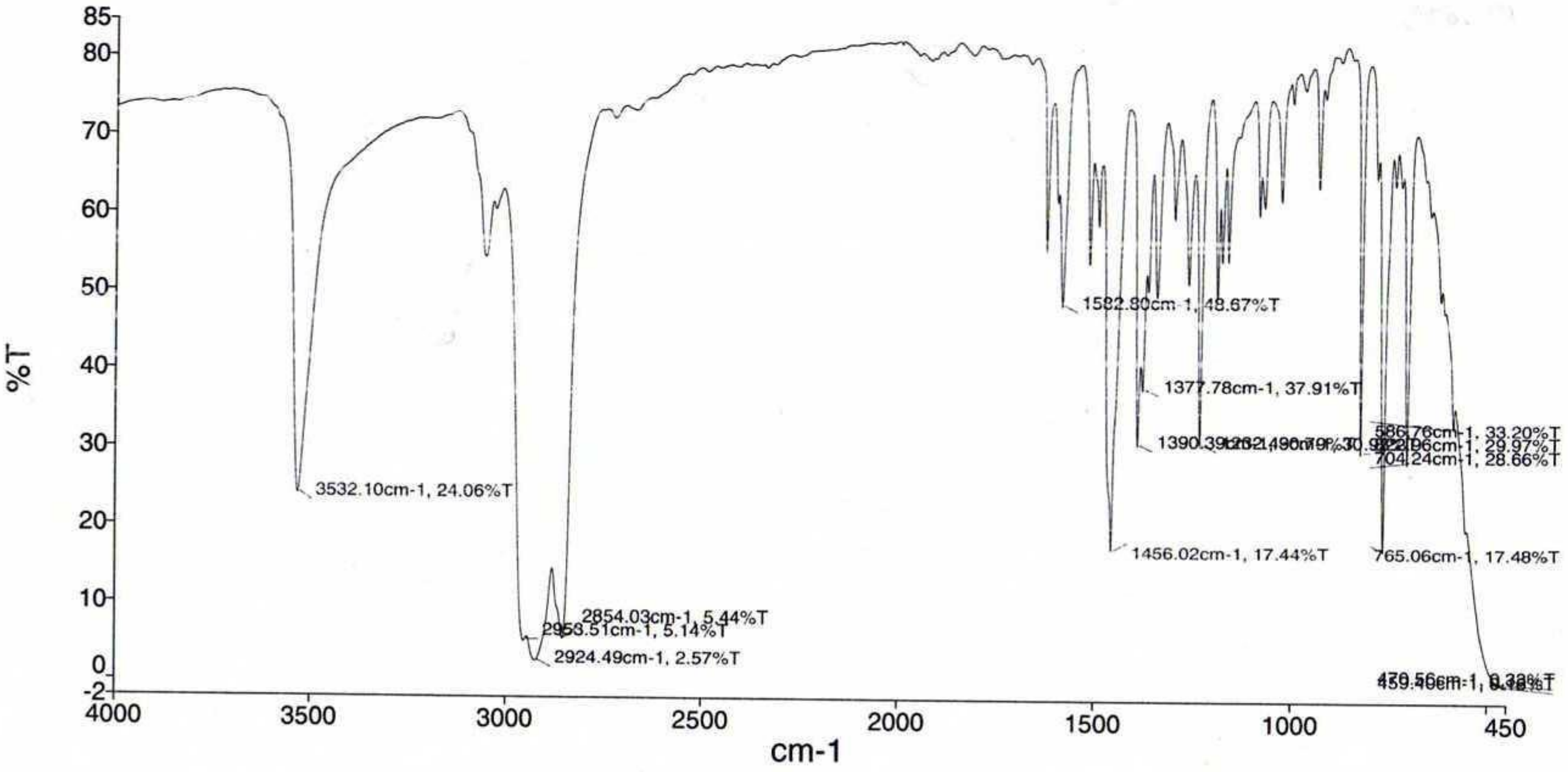

Administrator 12 Sample 012 By Administrator date Monday, January 26 2009

Page 1

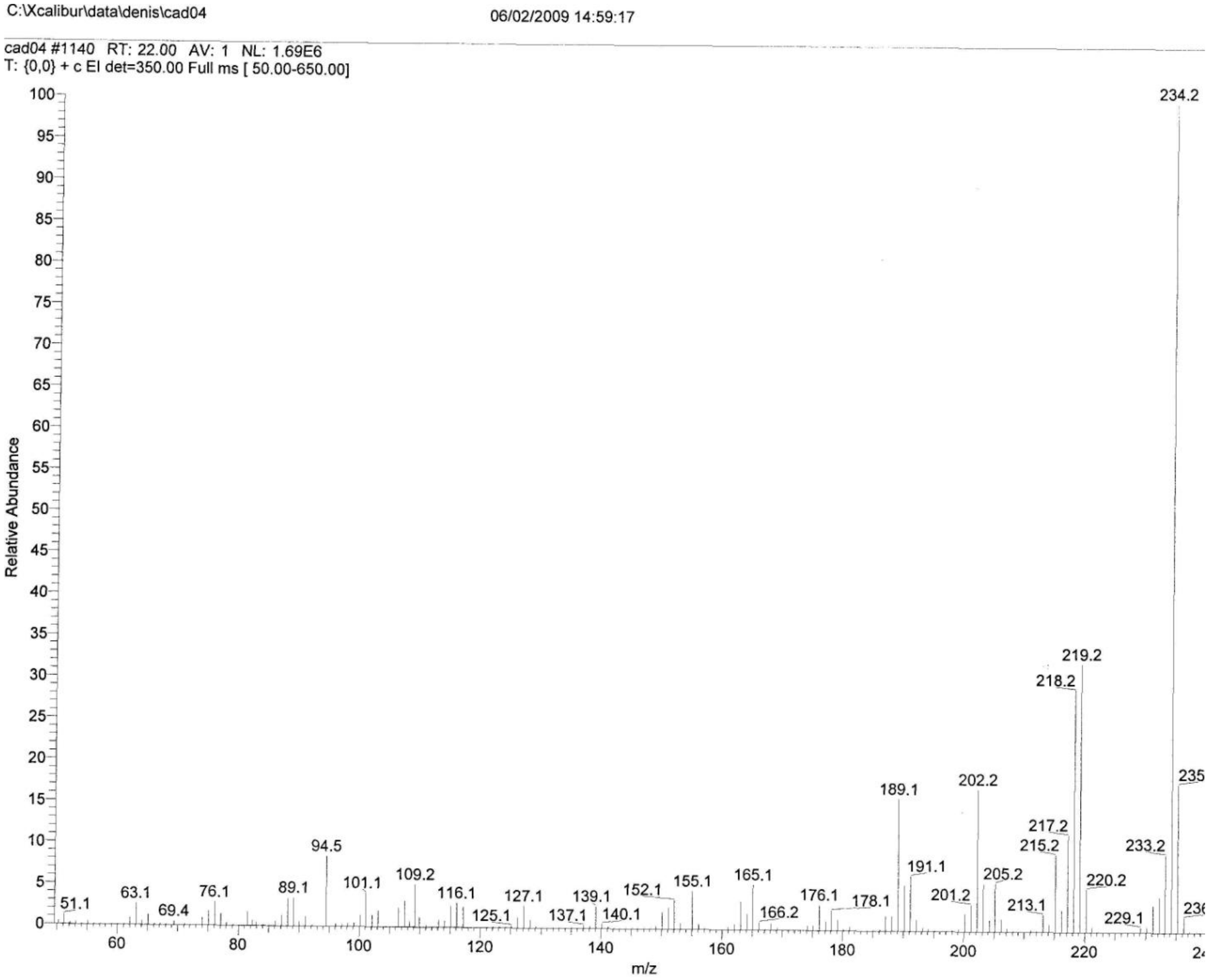
C:\Xcalibur\data\denis\cad04
06/02/2009 14:59:17
cad04 #1140 RT: 22.00 AV: 1 NL: 1.69E6
T: {0,0} + c EI det=350.00 Full ms [50.00-650.00]
Relative Abundance
m/z
234.2
219.2
218.2
202.2
189.1
235
217.2
215.2
233.2
191.1
205.2
220.2
94.5
109.2
101.1
89.1
76.1
63.1
51.1
69.4
116.1
125.1
127.1
137.1
139.1
140.1
152.1
155.1
165.1
166.2
176.1
178.1
201.2
213.1
229.1

Compound 5: 8-(4-methoxyphenyl)-1-naphthol

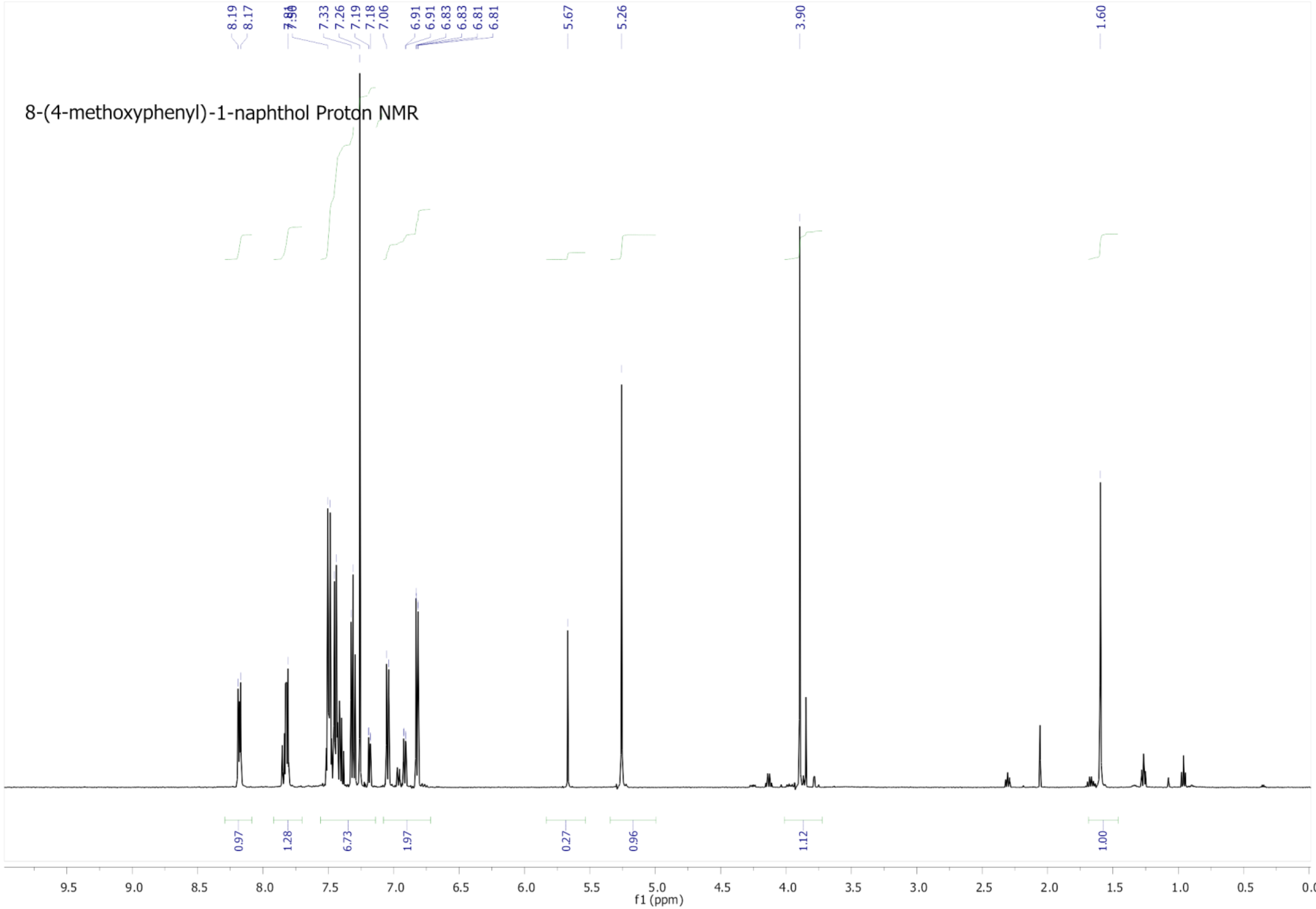

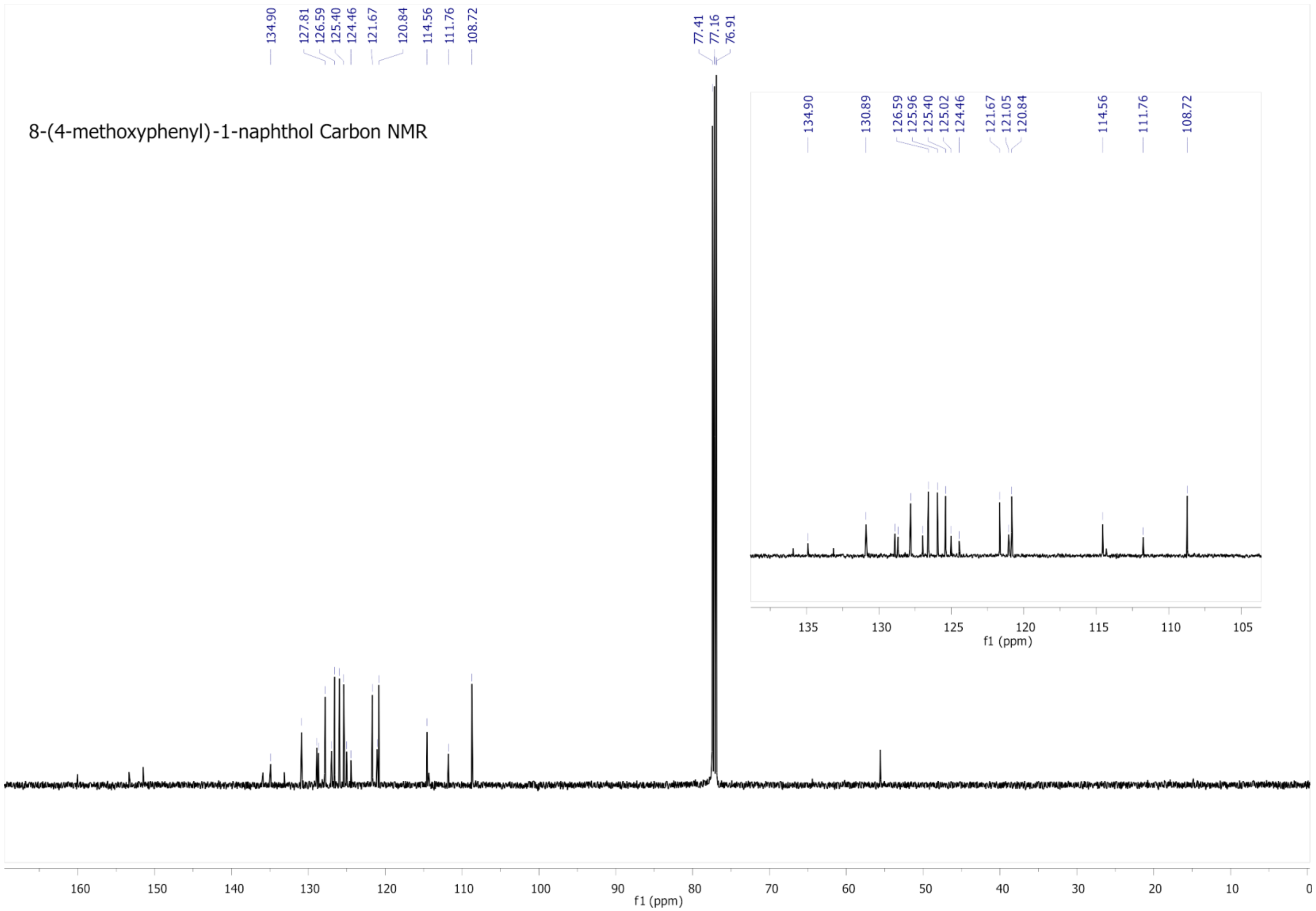

8-(4-methoxyphenyl)-1-naphthol Carbon NMR
134.90
127.81
126.59
125.40
124.46
121.67
120.84
114.56
111.76
108.72
77.41
77.16
76.91
130.89
125.96
125.02
121.05
f1 (ppm)

PerkinElmer Spectrum Express Version 1.02.00
26/01/2009 14:48

Analyst Administrator
Date 26 January 2009 14:48

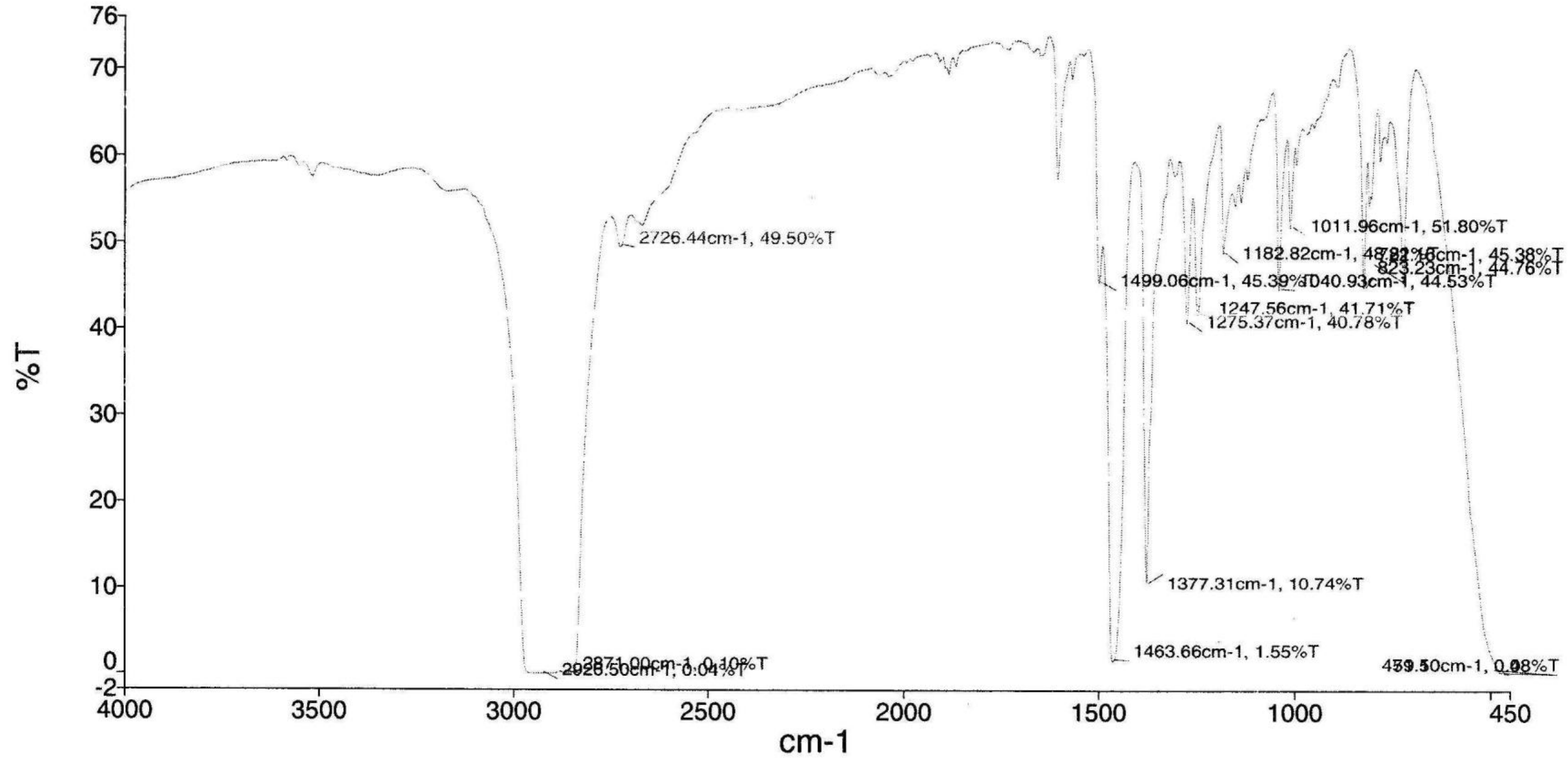

Administrator 11 Sample 011 By Administrator date Monday, January 26 2009

C:\Xcalibur\data\denis\cad03 06/02/2009 14:16:48

cad03 #1228-1230 RT: 23.47-23.50 AV: 3 SB: 11 23.25-23.42 NL: 1.47E4
T: {0,0} + c EI det=350.00 Full ms [50.00-650.00]

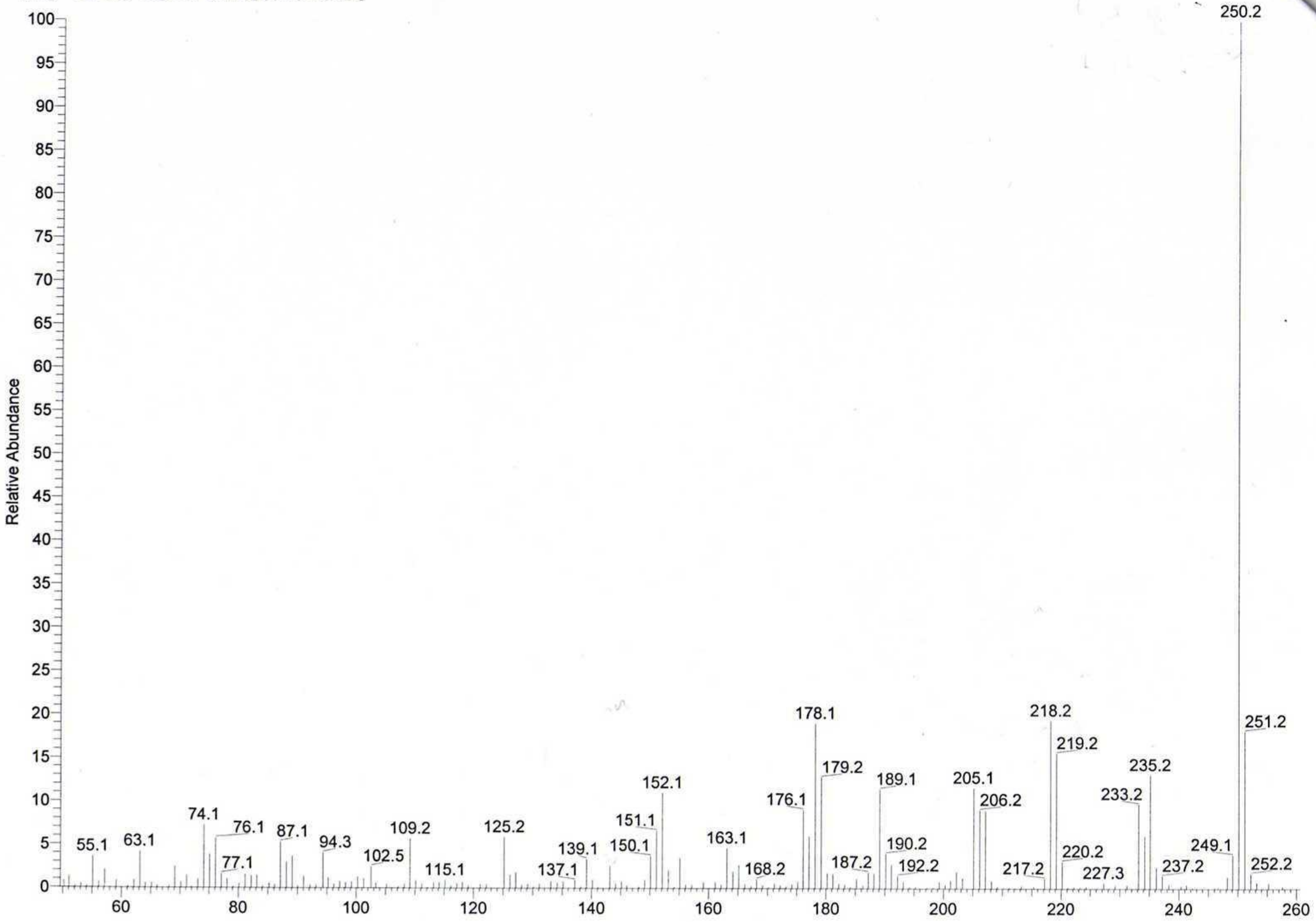

Supplement: File 2 — Original spectral data for compounds 1–5. [file Beilstein_J_Org_Chem-07-320-s002.pdf]
