# Supplementary material for: Anion–π interactions influence pKa values
Source: Beilstein J Org Chem. 2011 Mar 17;7:320–8. doi: 10.3762/bjoc.7.42 (PMC3079108; doi:10.3762/bjoc.7.42)

Supporting Information

for

Anion–π interactions influence pK_a values

Christopher J. Cadman and Anna K. Croft*

Address: School of Chemistry, University of Wales Bangor, Bangor, Gwynedd, LL57 2UW, United Kingdom. Fax: +44 1248 370 528. Tel: +44 1248 382 375.

Email: Anna K. Croft - a.k.croft@bangor.ac.uk

*Corresponding author

Molecular graphs for molecules 1(a)-5(a) and 8-12

generated with AIM2000

Naphthol derivatives:

1(a)

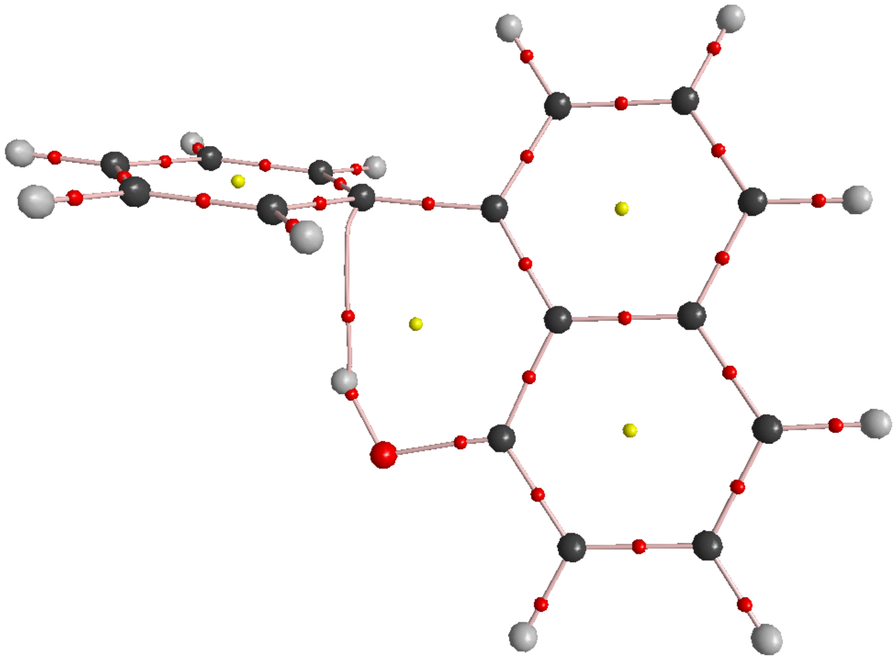

2(a)

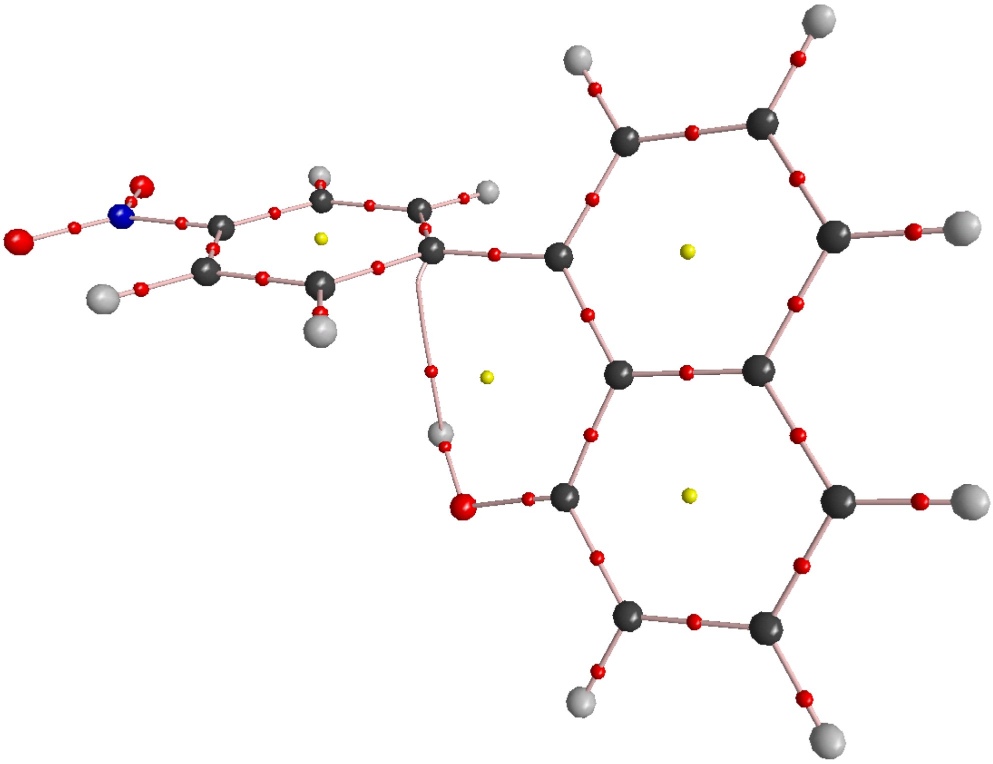

3(a)

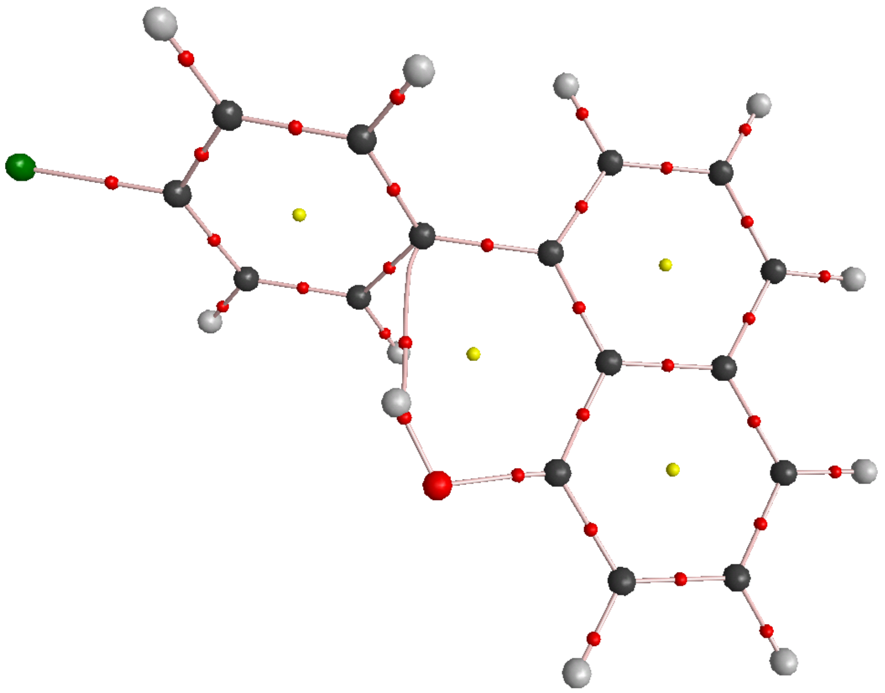

4(a)

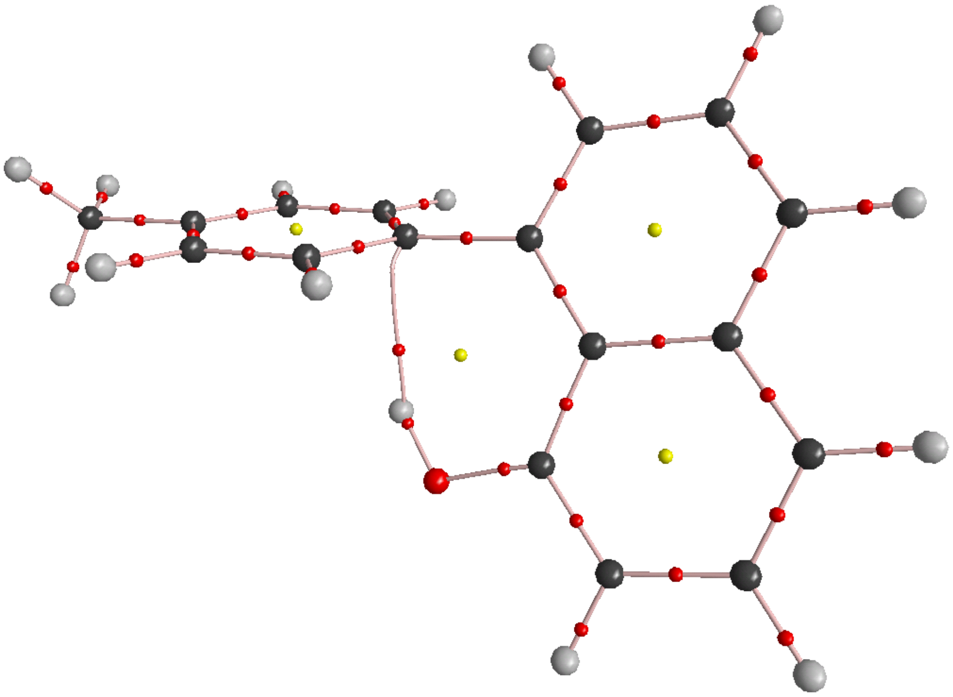

5(a)

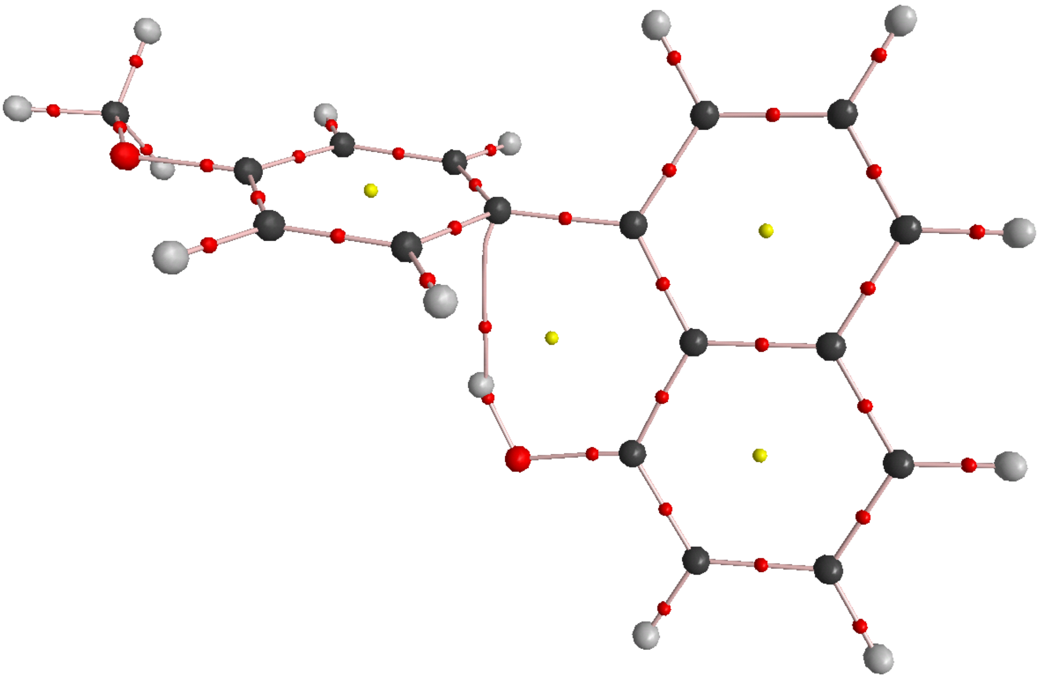

Anions:

8

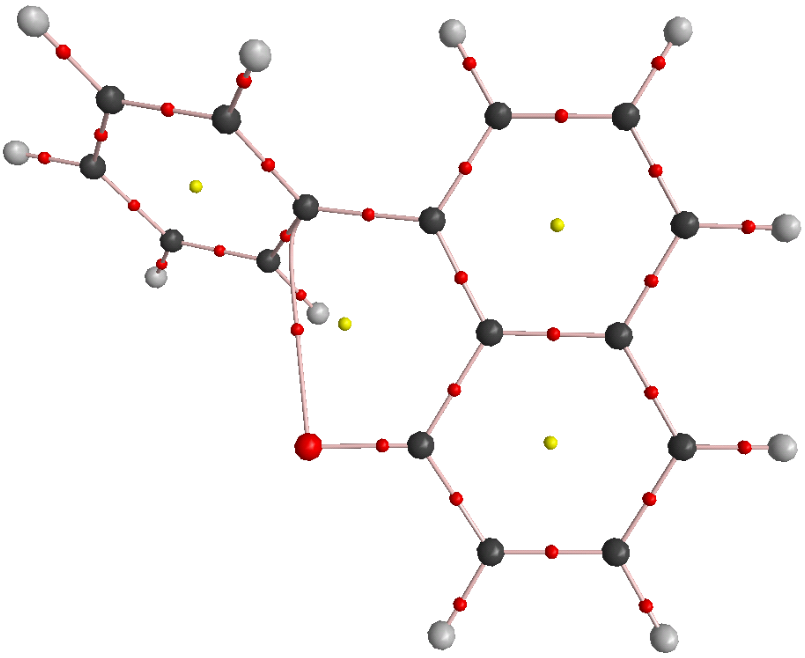

9

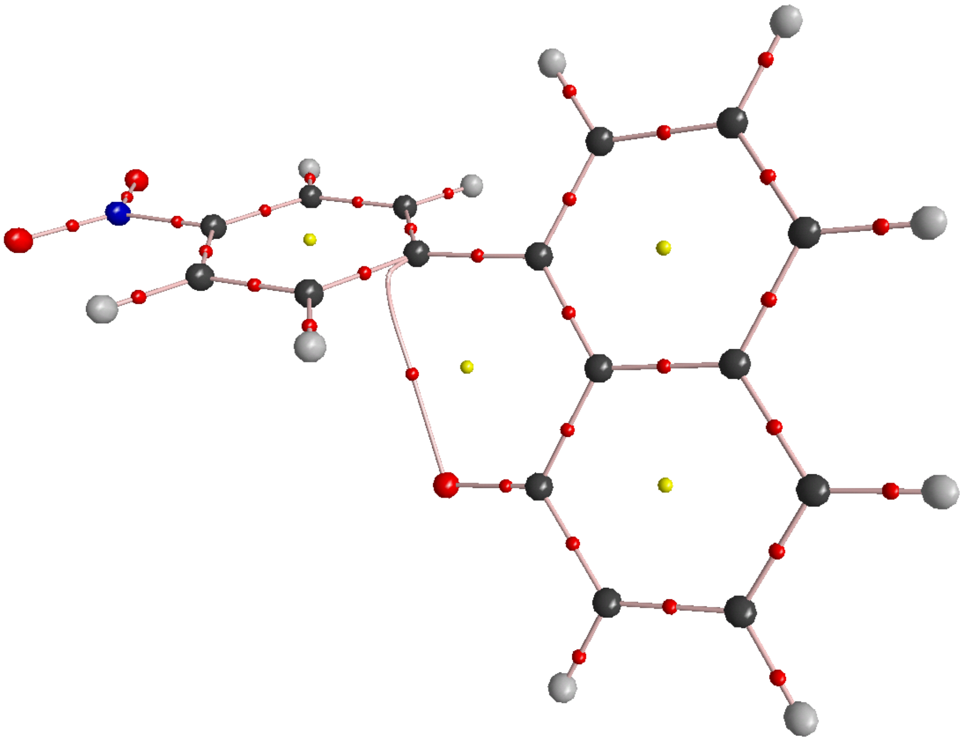

10

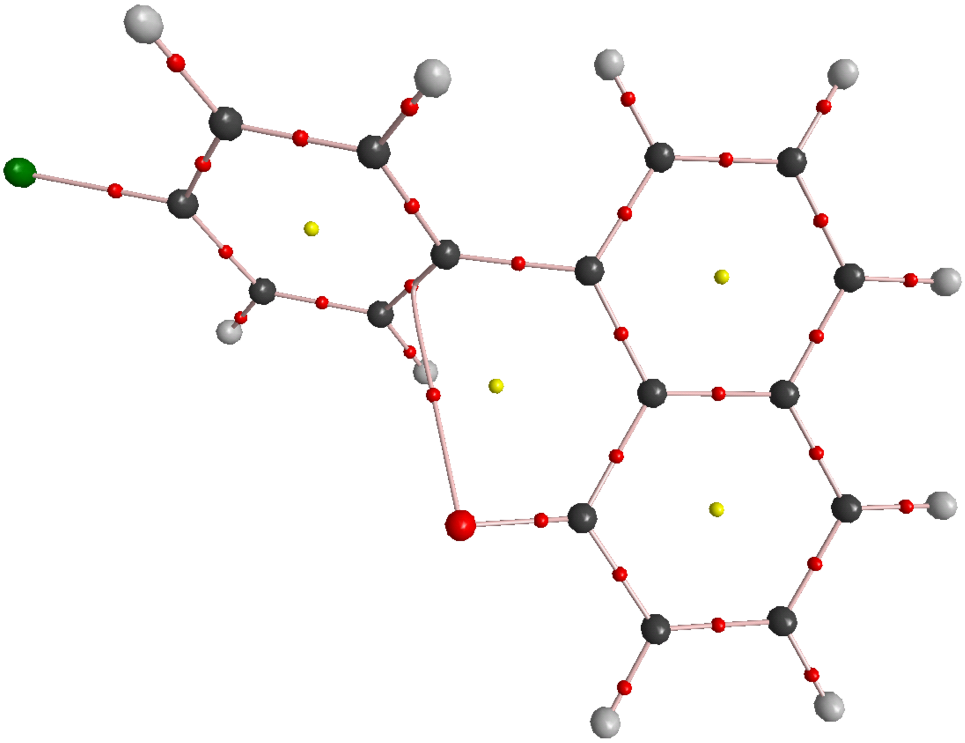

11

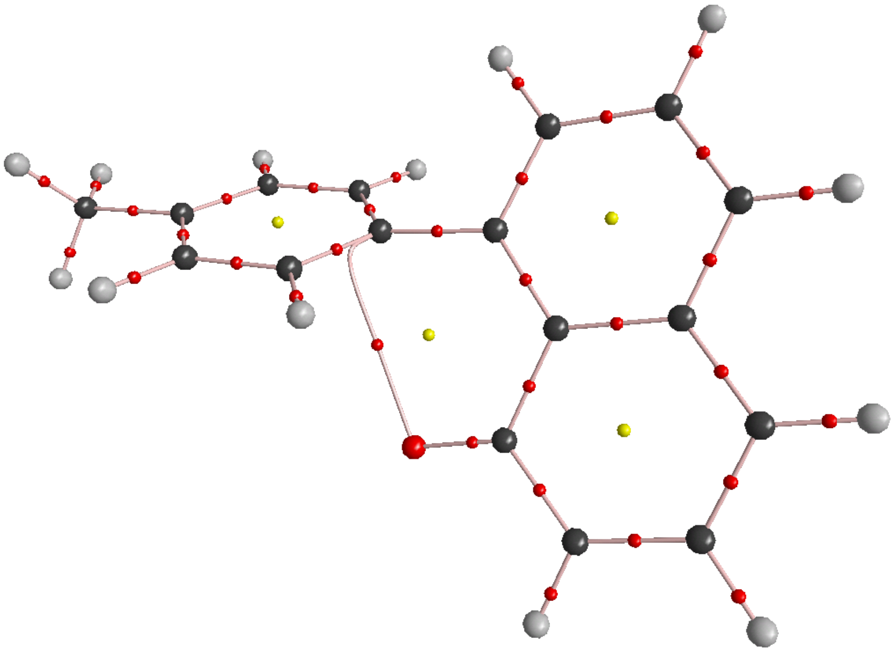

12

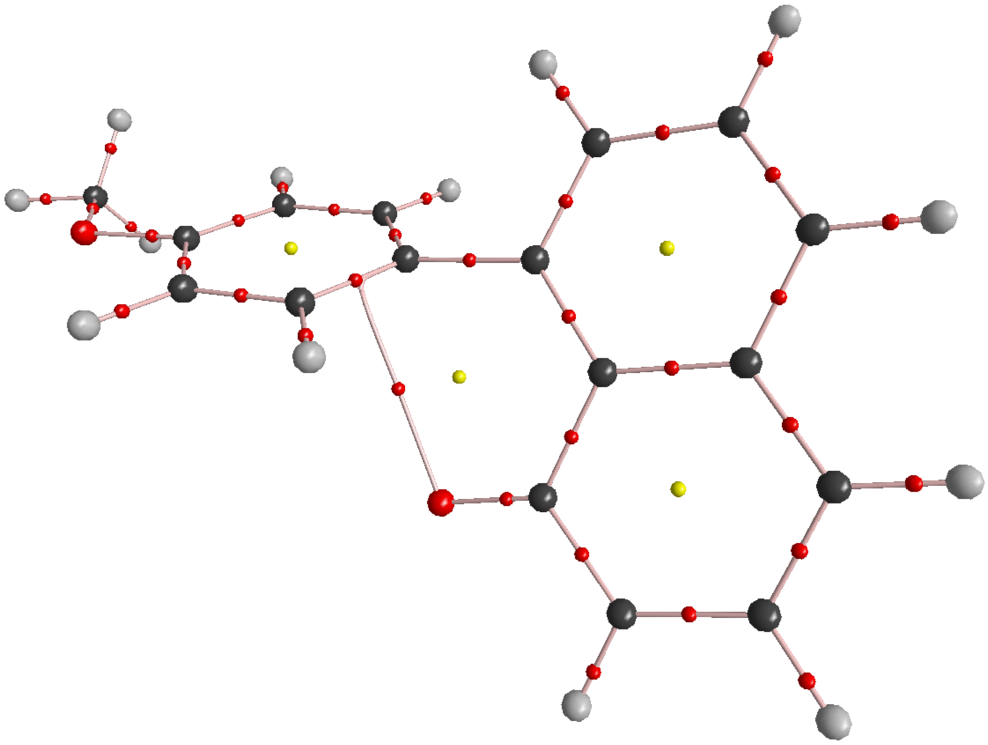

Supplement: File 3 — Molecular graphs for molecules 1(a)-5(a) and 8-12. [file Beilstein_J_Org_Chem-07-320-s003.pdf]
