# Supplementary material for: Anion–π interactions influence pKa values
Source: Beilstein J Org Chem. 2011 Mar 17;7:320–8. doi: 10.3762/bjoc.7.42 (PMC3079108; doi:10.3762/bjoc.7.42)
Supplement: File 6 — Crystal structure refinement details for compound 4. [file Beilstein_J_Org_Chem-07-320-s006.pdf]

Supporting Information

for

Anion–π interactions influence pK_a values

Christopher J. Cadman and Anna K. Croft*

Address: School of Chemistry, University of Wales Bangor, Bangor, Gwynedd, LL57 2UW, United Kingdom. Fax: +44 1248 370 528. Tel: +44 1248 382 375.

Email: Anna K. Croft - a.k.croft@bangor.ac.uk

*Corresponding author

Crystal structure refinement details for compound 4

Table 1: Crystal data and structure refinement details.

Identification code	**2009src0664**	
Empirical formula	$C_{17}H_{14}O$	
Formula weight	234.28	
Temperature	120(2) K	
Wavelength	0.71073 Å	
Crystal system	Monoclinic	
Space group	*C*2/*c*	
Unit cell dimensions	*a* = **13.3991(5) Å**	α = **90°**
	b = **8.2341(3) Å**	β = **96.356(2)°**
	c = **22.8014(8) Å**	γ = **90°**
Volume	2500.20(16) $Å^3$	
Z	8	
Density (calculated)	1.245 Mg / m^3	
Absorption coefficient	0.076 mm^{-1}	
F(000)	992	
Crystal	Block; colourless	
Crystal size	0.30 × 0.30 × 0.10 mm^3	
θ range for data collection	2.99 – 27.47°	
Index ranges	$-17 \le h \le 17$, $-10 \le k \le 10$, $-29 \le l \le 29$	
Reflections collected	17562	
Independent reflections	2874 [R_{int} = 0.0602]	
Completeness to θ = 27.47°	99.9 %	
Absorption correction	Semi–empirical from equivalents	
Max. and min. transmission	0.9925 and 0.9776	
Refinement method	Full-matrix least-squares on F^2	
Data / restraints / parameters	2874 / 0 / 165	
Goodness-of-fit on F^2	1.046	
Final *R* indices [$F^2 > 2\sigma(F^2)$]	*R1* = 0.0553, *wR2* = 0.1463	
R indices (all data)	*R1* = 0.0821, *wR2* = 0.1638	
Largest diff. peak and hole	0.270 and –0.284 e $Å^{-3}$	

Diffractometer: *Nonius KappaCCD* area detector (ϕ scans and ω scans to fill *asymmetric unit*). **Cell determination:** DirAx (Duisenberg, A.J.M.(1992). J. Appl. Cryst. 25, 92-96.) **Data collection:** Collect (Collect: Data collection software, R. Hooft, Nonius B.V., 1998). **Data reduction and cell refinement**: *Denzo* (Z. Otwinowski & W. Minor, *Methods in Enzymology* (1997) Vol. **276**: *Macromolecular Crystallography*, part A, pp. 307–326; C. W. Carter, Jr. & R. M. Sweet, Eds., Academic Press). **Absorption correction**: Sheldrick, G. M. SADABS - Bruker Nonius area detector scaling and absorption correction - V2.10 **Structure solution**: *SHELXS97* (G. M. Sheldrick, Acta Cryst. (1990) A**46** 467–473). **Structure refinement**: *SHELXL97* (G. M. Sheldrick (1997), University of Göttingen, Germany). **Graphics:** Cameron - A Molecular Graphics Package. (D. M. Watkin, L. Pearce and C. K. Prout, Chemical Crystallography Laboratory, University of Oxford, 1993).

Special details:

Table 2: Atomic coordinates [$\times 10^4$], equivalent isotropic displacement parameters [$Å^2 \times 10^3$] and site occupancy factors. U_{eq} is defined as one third of the trace of the orthogonalized U^{ij} tensor.

Atom	*x*	*y*	*z*	U_{eq}	*S.o.f.*
C1	6312(1)	3436(2)	6611(1)	25(1)	1
C2	5913(1)	4631(2)	6933(1)	30(1)	1
C3	6520(1)	5838(2)	7221(1)	33(1)	1
C4	7528(1)	5845(2)	7183(1)	32(1)	1
C5	9022(1)	4677(2)	6810(1)	30(1)	1
C6	9469(1)	3510(2)	6507(1)	31(1)	1
C7	8881(1)	2258(2)	6219(1)	28(1)	1
C8	7857(1)	2191(2)	6228(1)	23(1)	1
C9	7371(1)	3397(2)	6547(1)	22(1)	1
C10	7969(1)	4651(2)	6846(1)	26(1)	1
C11	7318(1)	805(2)	5907(1)	24(1)	1
C12	7393(1)	−760(2)	6144(1)	28(1)	1
C13	6936(1)	−2067(2)	5841(1)	29(1)	1
C14	6391(1)	−1873(2)	5288(1)	27(1)	1
C15	6319(1)	−315(2)	5048(1)	29(1)	1
C16	6772(1)	998(2)	5351(1)	28(1)	1
C17	5891(1)	−3307(2)	4971(1)	36(1)	1
O1	5648(1)	2296(2)	6376(1)	35(1)	1

Table 3: Bond lengths [Å] and angles [°].

C1–O1	1.362(2)
C1–C2	1.371(2)
C1–C9	1.443(2)
C2–C3	1.400(3)
C2–H2	0.9500
C3–C4	1.364(3)
C3–H3	0.9500
C4–C10	1.415(2)
C4–H4	0.9500
C5–C6	1.361(3)
C5–C10	1.423(2)
C5–H5	0.9500
C6–C7	1.414(2)
C6–H6	0.9500
C7–C8	1.376(2)
C7–H7	0.9500
C8–C9	1.429(2)
C8–C11	1.498(2)
C9–C10	1.432(2)
C11–C12	1.396(2)
C11–C16	1.402(2)
C12–C13	1.385(2)
C12–H12	0.9500
C13–C14	1.396(2)
C13–H13	0.9500
C14–C15	1.393(2)
C14–C17	1.503(2)
C15–C16	1.386(2)
C15–H15	0.9500
C16–H16	0.9500
C17–H17A	0.9800
C17–H17B	0.9800
C17–H17C	0.9800
O1–H1	0.8400
O1–C1–C2	115.46(15)
O1–C1–C9	123.42(14)
C2–C1–C9	121.10(16)
C1–C2–C3	121.31(16)
C1–C2–H2	119.3
C3–C2–H2	119.3
C4–C3–C2	120.06(16)
C4–C3–H3	120.0
C2–C3–H3	120.0
C3–C4–C10	120.49(16)
C3–C4–H4	119.8
C10–C4–H4	119.8
C6–C5–C10	120.92(16)
C6–C5–H5	119.5
C10–C5–H5	119.5
C5–C6–C7	119.77(16)
C5–C6–H6	120.1
C7–C6–H6	120.1
C8–C7–C6	121.87(16)
C8–C7–H7	119.1
C6–C7–H7	119.1
C7–C8–C9	119.41(15)
C7–C8–C11	116.79(14)
C9–C8–C11	123.79(14)
C8–C9–C10	118.75(14)
C8–C9–C1	125.15(15)
C10–C9–C1	116.07(14)
C4–C10–C5	119.79(16)
C4–C10–C9	120.95(15)
C5–C10–C9	119.26(15)
C12–C11–C16	117.60(15)
C12–C11–C8	120.24(14)
C16–C11–C8	122.07(14)
C13–C12–C11	120.98(15)
C13–C12–H12	119.5
C11–C12–H12	119.5
C12–C13–C14	121.34(15)
C12–C13–H13	119.3
C14–C13–H13	119.3
C15–C14–C13	117.88(15)
C15–C14–C17	121.80(16)
C13–C14–C17	120.32(16)
C16–C15–C14	120.96(15)
C16–C15–H15	119.5
C14–C15–H15	119.5
C15–C16–C11	121.23(16)
C15–C16–H16	119.4
C11–C16–H16	119.4
C14–C17–H17A	109.5
C14–C17–H17B	109.5
H17A–C17–H17B	109.5
C14–C17–H17C	109.5
H17A–C17–H17C	109.5
H17B–C17–H17C	109.5
C1–O1–H1	109.5

Symmetry transformations used to generate equivalent atoms:

Table 4: Anisotropic displacement parameters [Å^2× 10^3]. The anisotropic displacement factor exponent takes the form: $-2\pi^2[h^2a^{*2}U^{11} + \cdots + 2\,h\,k\,a^*\,b^*\,U^{12}]$.

Atom	U^{11}	U^{22}	U^{33}	U^{23}	U^{13}	U^{12}
C1	29(1)	25(1)	22(1)	4(1)	4(1)	0(1)
C2	34(1)	33(1)	25(1)	4(1)	9(1)	7(1)
C3	46(1)	30(1)	24(1)	−2(1)	8(1)	7(1)
C4	46(1)	27(1)	23(1)	−2(1)	2(1)	−2(1)
C5	33(1)	30(1)	27(1)	4(1)	0(1)	−9(1)
C6	23(1)	40(1)	31(1)	6(1)	3(1)	−5(1)
C7	26(1)	30(1)	30(1)	4(1)	7(1)	3(1)
C8	26(1)	22(1)	22(1)	3(1)	4(1)	0(1)
C9	27(1)	19(1)	19(1)	3(1)	3(1)	0(1)
C10	34(1)	23(1)	19(1)	4(1)	1(1)	−3(1)
C11	25(1)	23(1)	24(1)	−1(1)	6(1)	1(1)
C12	36(1)	26(1)	23(1)	2(1)	3(1)	−1(1)
C13	37(1)	21(1)	29(1)	0(1)	7(1)	0(1)
C14	25(1)	27(1)	29(1)	−6(1)	9(1)	−1(1)
C15	28(1)	34(1)	25(1)	−1(1)	2(1)	−1(1)
C16	31(1)	25(1)	27(1)	4(1)	4(1)	3(1)
C17	36(1)	33(1)	40(1)	−10(1)	7(1)	−8(1)
O1	27(1)	36(1)	43(1)	−9(1)	10(1)	−4(1)

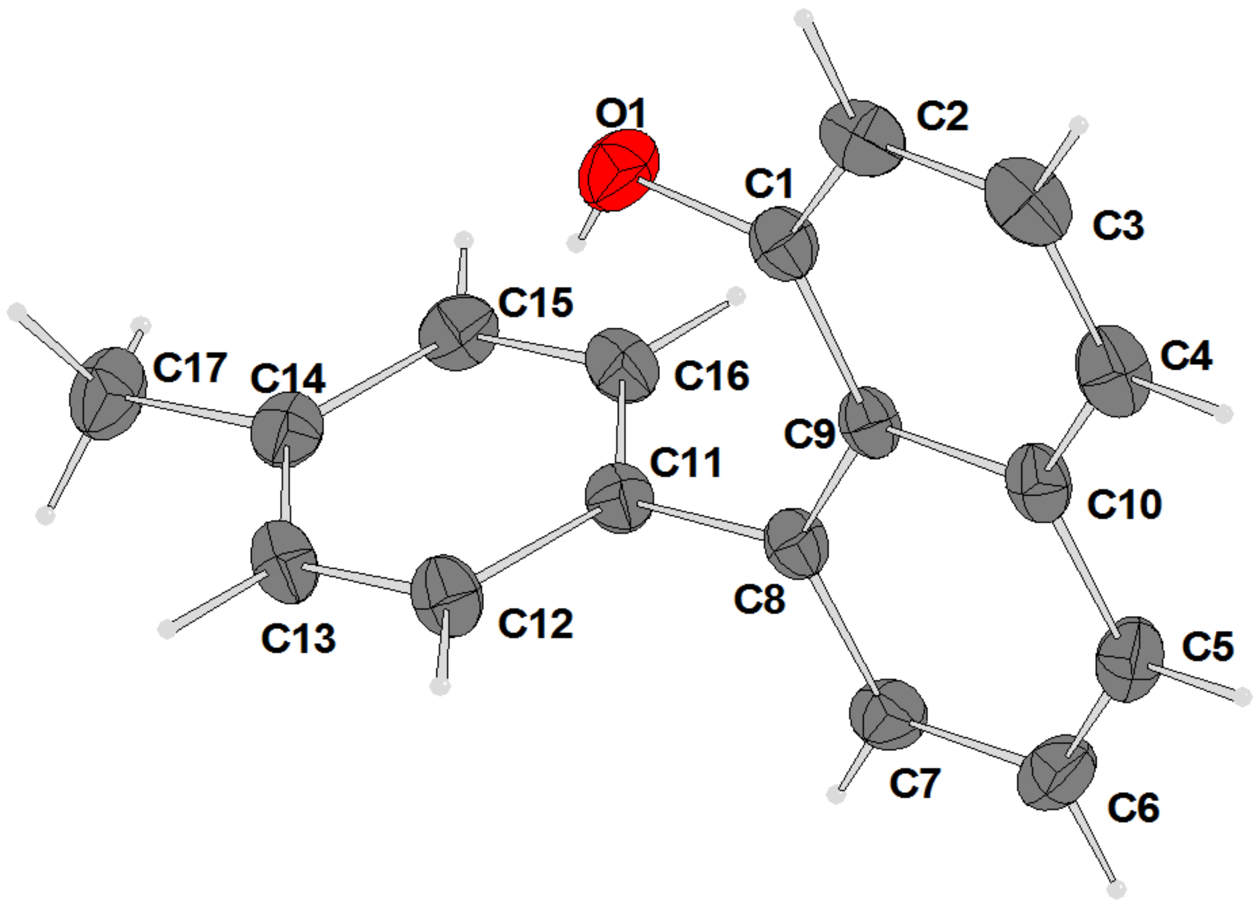

Thermal ellipsoids drawn at the 50% probability level.
